# A low-inflammatory diet is associated with a lower incidence of diabetes: role of diabetes-related genetic risk

**DOI:** 10.1186/s12916-023-03190-1

**Published:** 2023-12-05

**Authors:** Rongrong Yang, Jing Lin, Hongxi Yang, Michelle M. Dunk, Jiao Wang, Weili Xu, Yaogang Wang

**Affiliations:** 1 Public Health Science and Engineering College, Tianjin University of Traditional Chinese Medicine, Tianjin, China; 2 School of Public Health, Tianjin Medical University, Heping District, Qixiangtai Road 22, Tianjin, 300070 China; 3 Aging Research Center, Department of Neurobiology, Health Care Sciences and Society Karolinska Institutet and Stockholm University, Stockholm, Sweden; 4 School of Integrative Medicine, Tianjin University of Traditional Chinese Medicine, Tianjin, China

**Keywords:** A low-inflammatory diet, Type 2 diabetes, Prediabetes, Genetic risk, Cohort study

## Abstract

**Background:**

Whether a low-inflammatory diet relates to type 2 diabetes risk remains unclear. We examined the association between a low-inflammatory diet and risk of type 2 diabetes among normoglycemic and prediabetic participants. We also explored whether a low-inflammatory diet modifies genetic risk for type 2 diabetes.

**Methods:**

Among 142,271 diabetes-free UK Biobank participants (aged 39–72 years), 126,203 were normoglycemic and 16,068 were prediabetic at baseline. Participants were followed for up to 15 years to detect incident type 2 diabetes. At baseline, dietary intake was assessed with a 24-h dietary record. An inflammatory diet index (IDI) was generated based on high-sensitivity C-reactive protein levels and was a weighted sum of 34 food groups (16 anti-inflammatory and 18 pro-inflammatory). Participants were grouped into tertiles corresponding to inflammatory level (low, moderate, and high) based on IDI scores. Prediabetes at baseline was defined as HbA1c 5.7–6.4% in diabetes-free participants. Incident type 2 diabetes and age of onset were ascertained according to the earliest recorded date of type 2 diabetes in the Primary Care and Hospital inpatient data. A diabetes-related genetic risk score (GRS) was calculated using 424 single-nucleotide polymorphisms. Data were analyzed using Cox regression and Laplace regression.

**Results:**

During follow-up (median 8.40 years, interquartile range 6.89 to 11.02 years), 3348 (2.4%) participants in the normoglycemia group and 2496 (15.5%) in the prediabetes group developed type 2 diabetes. Type 2 diabetes risk was lower in normoglycemic (hazard ratio [HR] = 0.71, 95% confidence interval [CI] 0.65, 0.78) and prediabetic (HR = 0.81, 95% CI 0.73, 0.89) participants with low IDI scores compared to those with high IDI scores. A low-inflammatory diet may prolong type 2 diabetes onset by 2.20 (95% CI 1.67, 2.72) years among participants with normoglycemia and 1.11 (95% CI 0.59, 1.63) years among participants with prediabetes. In joint effect analyses, normoglycemic or prediabetes participants with low genetic predisposition to type 2 diabetes and low IDI scores had a significant 74% (HR = 0.26, 95% CI 0.21, 0.32) or 51% (HR = 0.49, 95% CI 0.40, 0.59) reduction in type 2 diabetes risk compared to those with high genetic risk plus high IDI scores. There were significant additive and multiplicative interactions between IDI and GRS in relation to type 2 diabetes risk in the normoglycemia group.

**Conclusions:**

A low-inflammatory diet is associated with a decreased risk of type 2 diabetes and may delay type 2 diabetes onset among participants with normal blood glucose or prediabetes. A low-inflammatory diet might significantly mitigate the risk of genetic factors on type 2 diabetes development.

**Supplementary Information:**

The online version contains supplementary material available at 10.1186/s12916-023-03190-1.

## Background

According to the International Diabetes Federation, 537 million adults (7.1% of the world’s population) in 2021 were living with diabetes, a figure predicted to rise to 783 million by 2045 [1]. Diabetes caused 6.7 million deaths in 2021, which is equivalent to one death every 5 s [2]. Prediabetes represents an intermediate state between normal blood glucose levels and clinical diabetes. Individuals with intermediate hyperglycemia are at increased risk of developing type 2 diabetes, but not everyone goes on to develop type 2 diabetes [3]. There is presently no cure for diabetes, and lifestyle modification with a healthy diet is regarded as the cornerstone of diabetes prevention, potentially conferring a 40–70% relative-risk reduction [4].

Accumulating evidence has shown that low-grade systemic inflammation plays a causal role in chronic diseases including type 2 diabetes, and dietary patterns are related to inflammation [5]. In recent years, a few studies have assessed diet quality based on its inflammatory potential and estimated the association between dietary inflammation and chronic disease [6–8]. Among the aforementioned studies, the most common inflammatory biomarker examined was high-sensitivity C-reactive protein (hsCRP) [8]. However, there is considerable variation in dietary habits across different populations, and characterization of dietary inflammation in a large European population has thus far been limited.

Currently, a few population-based studies have reported a significant association between dietary patterns with higher inflammatory potential and increased risk of type 2 diabetes [9, 10]. However, the lack of such an association in other studies raises questions [11, 12]. Additionally, one randomized controlled feeding study with a small sample failed to observe a significant association between an anti-inflammatory diet and prediabetes [13]. So far, no studies have evaluated the impact of an anti-inflammatory diet on the progression from prediabetes to diabetes.

Genetic and lifestyle-related factors may both contribute to the development of type 2 diabetes [14]. Moreover, the effect of genetic variants may change in response to alterations in the environment [15]. Previous studies have demonstrated that genetic variation and interplay between diet and genetic predisposition may account for considerable individual differences in response to dietary prevention of T2D [14–16]. Investigating gene-diet interactions in type 2 diabetes development therefore offers a unique opportunity to identify susceptible populations and determine to what extent they may benefit from personalized nutrition recommendations for type 2 diabetes prevention [16]. However, given the conflicting and limited research currently available, the question remains whether adherence to a low-inflammatory diet may mitigate genetic predisposition to type 2 diabetes.

In the current study, we sought to (1) calculate an inflammatory diet index (IDI) to assess dietary inflammatory potential; (2) examine the associations between a low-inflammatory diet and risk of type 2 diabetes among normoglycemic and prediabetic participants; and (3) investigate whether a low-inflammatory diet may mitigate diabetes-related genetic risk using data from the large population-based cohort study within the UK biobank.

## Methods

### Study population

**Fig. 1:**
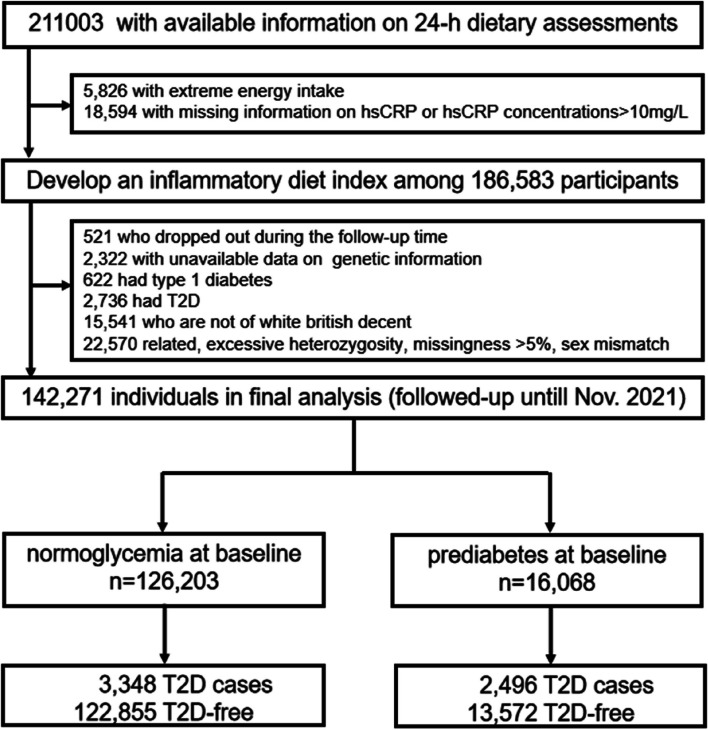
Flowchart of the study population. hsCRP, high-sensitivity C-reactive protein; T2D, type 2 diabetes

This large population-based prospective study included participants from the UK Biobank. From 2006 to 2010, 502,507 adults from 23 centers across England, Scotland, and Wales aged 40–70 years were invited to participate in a full-scale screening through touchscreen questionnaires and face-to-face interviews. Out of 211,003 individuals with at least one 24-h (24-h) dietary assessment, we excluded 5826 with extreme energy intake (men: <800 or >4200 kcal/days; women: <600 or >3500 kcal/days), and 18,594 who had missing information on hsCRP or hsCRP concentrations >10 mg/L. A total of 186,583 participants were available for the calculation of the IDI. Among them, we further excluded 521 individuals who dropped out during the follow-up period, 622 who had type 1 diabetes, 2736 who had type 2 diabetes, 2322 missing genetic data, 15,541 who were not of white British descent, and 22,570 who were related or had excessive heterozygosity, missingness >5%, or sex mismatch. In total, 142,271 participants were enrolled in the current analysis, consisting of a normoglycemia group (*n*=126,203) and a prediabetes group (*n*=16,068) (Figure 1).

All participants provided informed consent. The data collection procedures were approved by the North West Multi-centre Research Ethics Committee (Manchester, UK).

### Data collection

Information on sex, age, education, and socioeconomic status was collected through a touchscreen questionnaire and interview. Education was categorized as college or university, upper secondary, lower secondary, vocational, or other. Socioeconomic status was defined based on the Townsend deprivation index [17] (encompassing information on social class, employment, car availability, and housing) and categorized as low (highest quintile), middle (quintiles 2 to 4), or high (lowest quintile) [18]. Physical activity was divided into three levels: (1) active physical activity level was defined as having ≥150 min per week of moderate physical activity (MPA) or ≥75 min per week of vigorous physical activity (VPA) or ≥150 min per week of MPA and VPA; (2) intermediate physical activity level was defined as 1–149 min per week of MPA or 1–74 min per week of VPA or 1–149 min per week of MPA and VPA; (3) inactive physical activity level was defined as no MPA or VPA [19]. Body mass index (BMI) was calculated as weight (kg) divided by height squared (m^2^). Information on hsCRP and glycated hemoglobin A1c (HbA1c) levels were obtained from fasting blood samples collected from participants at the initial screening visit. Hypertension was defined as a self-reported history of hypertension, systolic blood pressure ≥140 mmHg, diastolic blood pressure ≥ 90 mmHg, or current use of anti-hypertensive drugs. We defined cardiovascular disease according to the International Classification of Diseases edition 10 (ICD-10): I20–I25 for coronary heart disease and I60–I64, I69 for stroke.

### Dietary assessment

Dietary data were collected at recruitment using the Oxford WebQ questionnaire, which was developed to acquire information on consumption of 206 types of foods and 32 types of drinks during the previous 24 h [20]. Moreover, participants who provided email addresses were invited to complete the Oxford WebQ for a total of four times every 3–4 months on variable days of the week during the follow-up period (online cycle 1, February 2011 to April 2011; online cycle 2, June 2011 to September 2011; online cycle 3, October 2011 to December 2011; online cycle 4, April 2012 to June 2012) [21]. Based on the study reported previously [22], food intake data in the current study were aggregated into 39 main groups aligned to the UK National Diet and Nutrition Survey and according to the similarity of their nutritional composition and culinary use. The mean daily quantity of each food consumed was calculated by multiplying the standard portion size of each food or drink by the amount consumed. We included participants with at least one 24-h dietary assessment in the current study. A sensitivity analysis among participants with at least two 24-h dietary assessments was also performed. Total energy intake was calculated using the UK Nutrient Databank food composition table [23].

### Assessment of inflammatory markers

Inflammation marker hsCRP was measured by an immunoturbidimetry method. Information on sample collection, processing, transport, and quality control has been reported previously [24]. Given that acute infection or medication use during blood sample collection may introduce bias, we excluded participants with hsCRP concentrations greater than 10 mg/L [25]. hsCRP concentration was ln-transformed due to skewed distribution.

### Assessment of prediabetes and diabetes

The HbA1c assay was performed using five Bio-Rad Variant II Turbo analyzers which underwent a rigorous validation protocol. These analyzers are manufactured by Bio-Rad Laboratories, Inc. and employ a High Performance Liquid Chromatography method.

Participants with baseline HbA1c levels between 5.7 and 6.4% (39–46 mmol/mol) were classified as prediabetic, and those with baseline HbA1c <5.7% (39 mmol/mol) were considered normoglycemic [26, 27]. Type 2 diabetes at baseline was ascertained according to self- and informant-reported history of diabetes, medical records (primary care, inpatient care in England/Scotland/ Wales [ICD-10: code E11], and the death registry), HbA1c ≥6.5% (48 mmol/mol), or fasting plasma glucose ≥126 mg/dl. Incident type 2 diabetes and age of onset were ascertained according to the earliest recorded date of type 2 diabetes diagnosis in the Primary Care and Hospital inpatient data.

### Genetic data and assessment of genetic risk score

Details of the quality control in the UK Biobank study has been summarized previously [28]. We excluded participants who self-reported ancestry other than white British, participants who were related (second degree or greater: kinship coefficient ≥0.884), those with high levels of heterozygosity and missingness (>5%), and participants whose reported sex was inconsistent with sex inferred from the genetic data. A weighted genetic risk score (GRS) for diabetes was calculated to assess the cumulative effect of genetic risk on type 2 diabetes. We selected 424 type 2 diabetes-associated risk variants (Additional file 1: Table S1) that have been identified previously based on the ancestry-specific analysis of Europeans in the largest genome-wide multiethnic meta-analysis [29]. We obtained the risk allele number of each single-nucleotide polymorphism (SNP) weighted by the effect size (β-coefficient) of these SNPs on type 2 diabetes from the European population in the latest genome-wide meta-analysis. An individual-level GRS was then calculated from the sum of the number of risk alleles present at each SNP weighted by the effect sizes from all SNPs included in the UK Biobank, which was produced using the PLINK “–score” command (Additional file 1: Method S1). The GRS was converted to a *Z*-score and divided into low, moderate, and high genetic risk tertiles.

### Statistical analysis

#### Identification of a low-inflammatory diet

As previously described [22], we generated the IDI based on the previous methods of constructing empirical dietary inflammatory pattern (EDIP) scores used in the Nurses’ Health Study and the Health Professionals Follow-up Study [7]. First, the mean daily intake of 39 food groups was calculated from the Oxford WebQ questionnaire (Additional file 1: Table S2). Second, a dietary pattern in relation to hsCRP concentration was obtained using reduced rank regression (RRR) (Additional file 1: Table S3). The first factor obtained from RRR with all 39 food groups was retained for subsequent analyses (we called this the RRR dietary pattern). The explained variations of the RRR dietary pattern for food groups and hsCRP are 3.9 and 2.7%, respectively. Third, stepwise linear regression was performed to identify the food groups which played an important role (*P*<0.05) in the RRR dietary pattern. Thirty-four food groups were retained, including 16 anti-inflammatory (nut, vegetarian protein alternative, starch, breakfast cereal, cheese, dessert, fish, wine, bread, fruit, pastry, vegetable, soup, tea, juice, coffee) and 18 pro-inflammatory foods (butter, organ meat, other alcohol, processed meat, red meat, other meat, ice cream, poultry, chocolate drink, low calorie drink, milk, egg, potato, snack, sweets, high calorie drink, smoothie, beer). Finally, the individual’s IDI score was calculated by weighting the sum of the intake of the filtered food groups based on the regression coefficients derived from the final stepwise linear regression (Additional file 1: Table S4). The specific formula is detailed in Additional file 1: Method S2. The IDI was operationalized as both a continuous (higher IDI score indicating more pro-inflammatory) and a categorical variable (low, moderate, and high tertiles; reference: high), with the low IDI tertile representing low-inflammatory diets. In the validation phase, we examined the association between IDI tertiles and hsCRP concentrations in test and retest subgroups using multivariable-adjusted linear regression models to calculate relative concentrations of hsCRP with the lowest tertile as reference (i.e., the ratios of hsCRP concentrations in the higher IDI tertiles to the concentration in the lowest tertile) [7, 8]. The test (*n*=130,608, 70% of 186,583 participants) and retest (*n*=55,975) in the divided subgroups indicated statistically significant and similar results (Additional file 1: Table S5).

#### Data analysis

The characteristics of participants by IDI group were compared using one-way analysis of variance/Kruskal-Wallis tests for continuous variables and chi-square tests for categorical variables.

Cox proportional hazards regression models were used to estimate hazard ratios (HRs) and 95% confidence intervals (CIs) for the incidence of type 2 diabetes according to IDI level in the normoglycemia and prediabetes groups. Follow-up time was calculated as the time from study entry to the first occurrence of type 2 diabetes, death, or final examination (November 31, 2021). The 50th percentile differences and 95% CIs for the time to type 2 diabetes development between different IDI levels were estimated using Laplace regression. All analyses were initially adjusted for age and sex, then further adjusted for education, Townsend deprivation index, smoking, physical activity, total energy intake, BMI, hypertension, cardiovascular disease, antidiabetic drug use, and GRS. For the models including the GRS, we additionally adjusted for the first 10 principal components of ancestry and genotyping batch.

The combined effect of a low-inflammatory diet and genetic background on type 2 diabetes risk was assessed by creating dummy variables based on joint exposures to both factors. The presence of an additive interaction was examined by estimating relative excess risk due to the interaction (RERI), the attributable proportion (AP), and the synergy index (SI). Additionally, we examined a multiplicative interaction by incorporating the two variables and their cross-product term in the same model.

Generalized structural equation modeling was performed to further test and quantify the mediation effect of hsCRP on the relationship between a low-inflammatory diet and type 2 diabetes. A bootstrapping method was used to estimate the 95% CI of indirect (mediated) effects. In this type of mediation analysis, mediation is confirmed if the bias-corrected 95% CI for the indirect effect does not include zero.

Missing values for education (*n*=591), Townsend deprivation index (*n*=156), smoking (*n*=365), physical activity (*n*=4867), and BMI (*n*=286) were imputed using chained equations (Markov chain Monte Carlo, MCMC). In sensitivity analysis, we calculated an IDI among participants with at least two 24-h dietary assessments (*n*=114,686). We additionally computed EDIP scores in our entire sample to corroborate our findings from IDI scores. The specific food items from the Oxford WebQ included in each food group for calculation of EDIP scores were determined based on those included by Tabung et al. [7] and are detailed in Additional file 1: Table S6. We then examined type 2 diabetes in relation to participants’ EDIP scores to compare to our primary analyses involving IDI scores. In addition, we repeated the main analyses: (1) after excluding missing values for covariates and (2) stratified by sex, age, or physical activity. Two-tailed *P*-values <0.05 were considered statistically significant. All statistical analyses were performed using SAS statistical software version 9.4 (SAS Institute, Cary, NC, USA) and Stata SE 15.0 for Windows (StataCorp).

## Results

### Characteristics of the study population at baseline

At baseline, the 142,271 diabetes-free participants (54.38% women; mean [SD] age 57.12 [7.54] years) included 126,203 individuals with normoglycemia and 16,068 prediabetes cases. Baseline characteristics of the study population by incident type 2 diabetes in the normoglycemia and prediabetes groups are presented in Table 1.

**Table 1:** Characteristics of the study population by incident type 2 diabetes (T2D) in normoglycemia and prediabetes groups

Characteristic	Normoglycemia group (*N* = 126,203)			Prediabetes group (*N* = 16,068)		
	T2D-free (*n* = 122,855)	T2D (*n* = 3348)	*P*	T2D-free (*n* = 13,572)	T2D (*n* = 2496)	*P*-value
Age	55.9 ± 7.8	59.32 ± 7.1	< 0.001	59.8 ± 6.4	60.5 ± 6.3	< 0.001
Female	67,772 (55.2)	1181 (35.3)	< 0.001	7488 (55.2)	932 (37.3)	< 0.001
Townsend deprivation index	− 1.9 ± 2.7	− 1.4 ± 2.9	< 0.001	− 1.8 ± 2.8	− 1.4 ± 2.9	< 0.001
Education			< 0.001			< 0.001
College or University degree	52,798 (43.0)	1005 (30.0)		4905 (36.1)	684 (27.4)	
Upper secondary	16,542 (13.5)	400 (11.9)		1683 (12.4)	314 (12.6)	
Lower secondary	31,507 (25.6)	930 (27.8)		3699 (27.2)	666 (26.7)	
Vocational	6501 (5.3)	270 (8.1)		830 (6.1)	237 (9.5)	
Other	15,507 (12.6)	743 (22.2)		2455 (18.1)	595 (23.8)	
Smoking status			< 0.001			< 0.001
Never	72,001 (58.6)	1491 (44.5)		6967 (51.3)	1049 (42.0)	
Previous	42,588 (34.7)	1508 (45.0)		5154 (38.0)	1207 (48.4)	
Current	8266 (6.7)	349 (10.5)		1451 (10.7)	240 (9.6)	
Physical activity			< 0.001			< 0.001
Unfavorable	11,790 (9.6)	574 (17.1)		1,473 (10.9)	369 (14.8)	
Intermediate	82,273 (67.0)	2160 (64.5)		9149 (67.4)	1667 (66.8)	
Favorable	28,792 (23.4)	614 (18.3)		2950 (21.7)	460 (18.4)	
Antidiabetic drug			< 0.001			< 0.001
No	121,503 (98.9)	2418 (72.2)		13,331 (98.2)	2144 (85.9)	
Yes	1,352 (1.1)	930 (27.8)		241 (1.8)	352 (14.1)	
Total energy intake, kcal/day	2077.8 ± 606.8	2119.1 ± 648.2	< 0.001	2094.9 ± 611.5	2130.0 ± 651.0	0.009
Genetic risk			< 0.001			< 0.001
Low	41,477 (33.8)	590 (17.6)		4701 (34.6)	655 (26.2)	
Moderate	41,086 (33.4)	987 (29.5)		4554 (33.6)	802 (32.1)	
High	40,292 (32.8)	1771 (52.9)		4317 (31.8)	1039 (41.6)	
Body mass index (kg/m^2^)	26.4 ± 4.2	30.9 ± 5.4	< 0.001	28.0 ± 4.7	31.2 ± 5.2	< 0.001
Hypertension			< 0.001			< 0.001
No	95,877 (78.0)	988 (29.5)		9046 (66.7)	685 (27.4)	
Yes	26,978 (22.0)	2360 (70.5)		4526 (33.3)	1811 (72.6)	
Cardiovascular disease			< 0.001			< 0.001
No	116,936 (95.2)	2912 (87.0)		12,339 (90.9)	2103 (84.3)	
Yes	5919 (4.80)	436 (13.0)		1233 (9.1)	393 (15.7)	

Data are presented as mean ± SD, median (IQR), or *n* (%)

### IDI and type 2 diabetes in the normoglycemia and prediabetes groups

During follow-up (median 8.40 years, interquartile range [IQR] 6.89 to 11.02 years), 3348 (2.7%) participants developed type 2 diabetes in the normoglycemia group and 2496 (15.5%) developed type 2 diabetes in the prediabetes group.

**Fig. 2:**
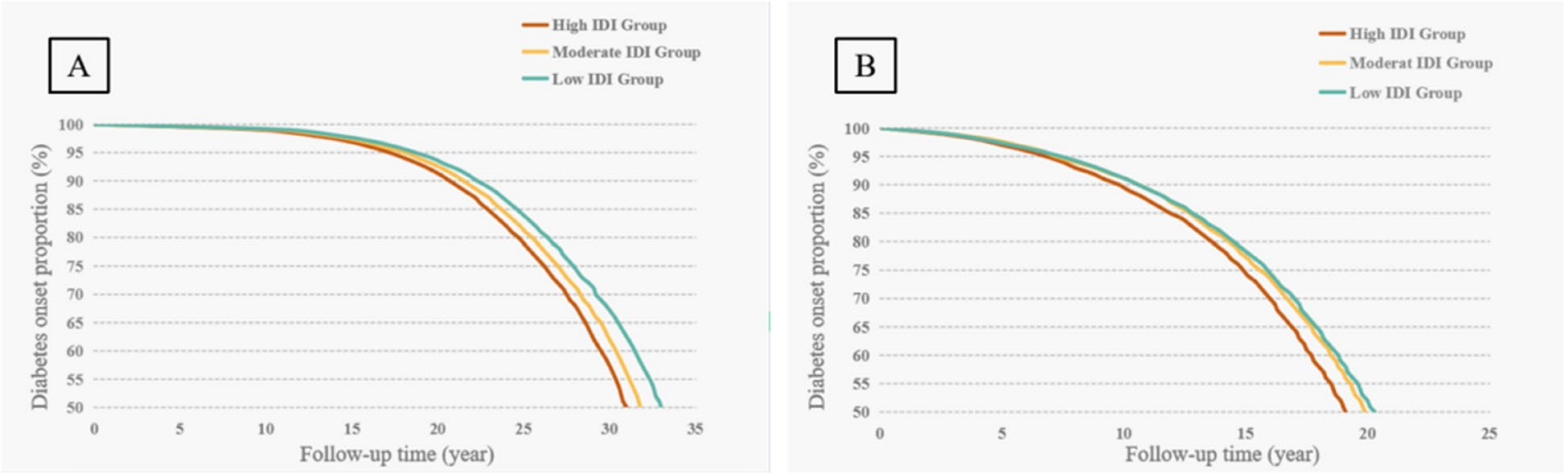
Laplace regression analysis for 50th percentile differences in year of type 2 diabetes onset by inflammatory diet index (IDI) indicator in normoglycemia (**A**) and prediabetes groups (**B**). The model was adjusted for age, sex, education, Townsend deprivation index, energy intake, smoking, physical activity, body mass index, hypertension, cardiovascular disease, antidiabetic drug, and genetic risk score

In the normoglycemia group, higher IDI scores (when treated as a continuous variable) were associated with a higher risk of type 2 diabetes (HR=1.16, 95% CI 1.12, 1.20) in multi-adjusted Cox regression models. Low and moderate IDI scores were associated with a decreased risk of type 2 diabetes (HR=0.71, 95% CI 0.65, 0.78; HR=0.86, 95% CI 0.79, 0.93, respectively) compared to high IDI scores. Laplace regression analysis showed that diets with low or moderate IDI scores delayed type 2 diabetes onset by 2.20 (95% CI 1.67, 2.72) and 1.07 (95% CI 0.63, 1.51) years, respectively, compared to high IDI scores (Table 2, Figure 2A, and Additional file 1: Table S7).

**Table 2:** Multi-adjusted hazard ratios (HRs), 50th percentile differences (PDs, years), and 95% confidence intervals (95% CIs) of incident type 2 diabetes (T2D) in relation to a low-inflammatory diet in normoglycemia and prediabetes groups

Inflammatory diet index	T2D in the normoglycemia group			T2D in the prediabetes group		
	No. of cases	Multi-adjusted HR (95% CI) ^**a**^	Multi-adjusted 50th PDs (95% CI)^**a**^	No. of cases	Multi-adjusted HR (95% CI)^**a**^	Multi-adjusted 50th PDs (95% CI)^**a**^
Continuous (per 1 SD increase)	3348	1.16 (1.12, 1.20)	− 0.94 (− 1.14, − 0.74)	2496	1.05 (1.01, 1.10)	− 0.37 (− 0.60, − 0.15)
Categorical						
High	1686	1.00 (Reference)	0.00 (Reference)	1115	1.00 (Reference)	0.00 (Reference)
Moderate	1008	0.86 (0.79, 0.93)	1.07 (0.63, 1.51)	769	0.87 (0.79, 0.96)	0.71 (0.23, 1.18)
Low	654	0.71 (0.65, 0.78)	2.20 (1.67, 2.72)	612	0.81 (0.73, 0.89)	1.11 (0.59, 1.63)

^**a**^Adjusted for age, sex, education, Townsend deprivation index, energy intake, smoking, physical activity, body mass index, hypertension, cardiovascular disease, antidiabetic drug, and genetic risk score

Among participants with prediabetes, continuous IDI scores were dose-dependently associated with type 2 diabetes, with each 1 SD increase in IDI score contributing to a 5% increased risk of type 2 diabetes. Low and moderate IDI scores were associated with a reduction in type 2 diabetes risk (HR=0.81, 95% CI 0.73, 0.89; HR=0.87, 95% CI 0.79, 0.96, respectively) compared to high IDI scores. Laplace regression analysis showed that compared to high IDI scores, low and moderate IDI scores delayed type 2 diabetes onset by 1.11 (95% CI 0.59, 1.63) and 0.71 (95% CI 0.23, 1.18) years, respectively (Table 2, Figure 2B, and Additional file 1: Table S7).

### Association between genetic risk and type 2 diabetes

Normoglycemic and prediabetic participants with moderate genetic risk for type 2 diabetes had higher type 2 diabetes incidence (HR=1.60, 95% CI 1.45, 1.77; HR=1.26, 95% CI 1.14, 1.40, respectively) compared to those with low genetic predisposition. Normoglycemic and prediabetic individuals with high type 2 diabetes genetic predisposition also had higher type 2 diabetes incidence (HR=2.59, 95% CI 2.35, 2.84; HR=1.69, 95% CI 1.53, 1.86, respectively) compared to those with low genetic predisposition (Additional file 1: Table S8).

### Joint effect of IDI and genetic risk score on type 2 diabetes risk

**Fig. 3:**
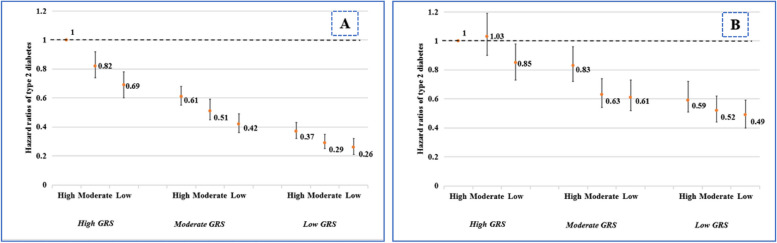
Multi-adjusted hazard ratios (HRs) and 95% confidence intervals (95% CIs) of type 2 diabetes (T2D) in relation to joint exposure of inflammatory diet index (IDI) and genetic risk score (GRS) in normoglycemia (**A**) and prediabetes (**B**) groups. The models were adjusted for age, sex, education, Townsend deprivation index, energy intake, smoking, physical activity, body mass index, hypertension, cardiovascular disease, antidiabetic drug, genetic risk score, the first 10 principal components of ancestry, and genotyping batch

Joint effect analyses revealed that among normoglycemic participants with low type 2 diabetes genetic predisposition, moderate and low IDI scores were associated with a significant 71% (HR=0.29, 95% CI 0.25, 0.35) and 74% (HR=0.26, 95% CI 0.21, 0.32) reduction, respectively, in type 2 diabetes risk compared to those with high genetic risk plus high IDI scores (Figure 3 and Additional file 1: Table S9). In stratified analyses, low or moderate IDI scores were associated with 17 or 34% lower type 2 diabetes risk among individuals with high genetic risk (Additional file 1: Table S10). There was a significant additive interaction between IDI score and genetic risk score on type 2 diabetes risk (RERI=0.184, 95% CI 0.183, 0.186; AP=0.064, 95% CI 0.063, 0.068; SI=1.110, 95% CI 1.108, 1.112) (Additional file 1: Table S11). There was also a significant multiplicative interaction between genetic risk score and IDI score on type 2 diabetes risk (HR=1.10, 95% CI 1.04, 1.24, *P*= 0.021).

Among prediabetic participants with low genetic risk, low IDI scores were associated with a significant 51% (OR=0.49, 95% CI 0.40, 0.59) decrease in type 2 diabetes risk compared to those with high genetic risk plus high IDI scores (Additional file 1: Table S9). Further joint analysis suggested non-significant additive and multiplicative effects between IDI score and genetic risk score on type 2 diabetes risk (additive: RERI=−0.118, 95% CI −0.367, 0.130; AP=−0.065, 95% CI −0.201, 0.071; SI=0.873, 95% CI 0.670, 1.137; multiplicative: HR=1.02, 95% CI 0.96, 1.09; *P*=0.466) (Additional file 1: Table S11).

### Supplementary analysis

We generated an IDI among participants with at least two 24-h dietary assessments, and the results were not very different than those from initial analyses among participants with at least one 24-h dietary assessment (Additional file 1: Table S12). We examined the relationship between participants’ EDIP scores and type 2 diabetes, and the results were not meaningfully altered (Additional file 1: Table S13). The results were also generally similar to those in the main analyses conducted after excluding missing values for covariates (Additional file 1: Table S14). Given that sex, age and physical activity may contribute to the development of systemic inflammation and type 2 diabetes, we performed stratified analyses, and the associations between IDI and type 2 diabetes risk did not vary by sex, age, and physical activity (Additional file 1: Table S15, S16 and S17). We also assessed the mediating role of hsCRP in the association between IDI and type 2 diabetes. In mediation analysis, a higher IDI was associated with an increased risk of type 2 diabetes (*β*= 0.0038, 95% CI 0.0029-0.0047). The association became slightly weaker when hsCRP was entered into the model (*β* = 0.0035, 95% CI 0.0026–0.0045), and hsCRP mediated about 7.10% of the association between IDI and type 2 diabetes (Additional file 1: Table S18 and Figure S1).

## Discussion

In this large-scale, nationwide prospective study of type 2 diabetes-free adults consisting of normoglycemic and prediabetic individuals, we found that (1) low IDI score (calculated from 16 anti-inflammatory and 18 pro-inflammatory foods) was dose-dependently associated with decreased type 2 diabetes risk; (2) a low-inflammatory diet may delay type 2 diabetes onset by about 2 years among participants with normoglycemia and 1.2 years among prediabetic participants; and (3) a low-inflammatory diet might significantly mitigate the risk of genetic factors on type 2 diabetes development.

A few previous studies have reported inflammation-based dietary patterns in different populations [6–8, 30]. Given that dietary habits vary across populations and the limited investigation of dietary inflammation in European populations, we calculated an IDI using a data-driven method to predict chronic low-grade systemic inflammation in approximately 190,000 European participants. Shivappa et al. developed the dietary inflammatory index (DII) that accounted for 45 pro- and anti-inflammatory food parameters, most of which are specific micronutrients and macronutrients (such as vitamin B12, vitamin C, protein, and n-3 fatty acids) rather than whole foods [6, 31]. However, the DII is difficult for the general public to easily understand and directly make use of given that we consume foods (consisting of numerous and interacting micro- and macronutrients) rather than isolated nutrients. Our IDI identified some key anti-inflammatory (nuts, vegetables, fruits, wine, coffee) and pro-inflammatory foods (red meat, processed meat, organ meet, sugar-sweetened beverages) which are consistent with previous studies involving food-based indices of dietary inflammation in other populations, such as the Anti-Inflammatory Diet Index created in a Nordic population (women, *n*=3503) [8, 32], the EDIP scores developed and validated in the US population [7], and a dietary inflammatory potential score constructed in the Chinese population [30]. Despite variations across studies in dietary habits and the type of inflammatory markers examined, the considerable overlap of our findings with previous reports underscores the involvement of these foods in modulating chronic inflammation.

Chronic low-grade inflammation substantially contributes to the development of type 2 diabetes [33, 34], and diet is one of the modifiable lifestyle-related factors that might partially modulate inflammation [6, 35]. Few studies to date have examined the relationship between dietary patterns with higher inflammatory potential and type 2 diabetes risk [9, 10], and findings have been inconsistent [11]. A previous longitudinal study from the USA has documented that higher dietary inflammatory potential was strongly related to an increased risk of type 2 diabetes among participants in the Nurses’ Health Study and the Health Professionals Follow-up Study [9]. Laouali et al. demonstrated that diets with elevated anti-inflammatory potential were associated with a lower risk of type 2 diabetes in a French prospective cohort of women [10]. A cross-sectional study using the dietary inflammatory index reported a positive association between a pro-inflammatory diet and type 2 diabetes among 1174 adult Mexicans [36]. By contrast, one Dutch cross-sectional study with a limited sample (*n*=1024) found that the adapted dietary inflammatory index was not significantly associated with glycated hemoglobin [11]. Another cross-sectional investigation among Iranian adults also documented non-significant associations between a pro-inflammatory diet and risk of insulin resistance [12]. Moreover, only one randomized controlled feeding study conducted in Portland, Oregon, with a small sample (intervention group: *n*=20) failed to observe improved blood sugar levels among prediabetic participants following a 6-week anti-inflammatory diet developed by scientists and naturopathic physicians [13]. Thus, evidence on the relationship between low-inflammatory diets and type 2 diabetes has remained unclear. In the present study, we found that diets lower in inflammation were associated with lower risk of type 2 diabetes among both normoglycemic and prediabetic participants. To the best of our knowledge, this is the first study to demonstrate that a low-inflammatory diet may delay the progression from prediabetes to type 2 diabetes.

It is widely known that type 2 diabetes is a complex genetic and lifestyle-related disorder [37]. Previously published genetic studies have demonstrated diet may differentially affect type 2 diabetes risk depending on an individual’s genetic risk [38]. Thus, the consequences of adhering to a healthy diet are considerably complex. A previous prospective study including 357,419 UK Biobank participants reported that higher diet quality (evaluated based on 10 foods predictive of type 2 diabetes risk) was significantly associated with greater reductions in blood HbA1c levels and type 2 diabetes risk among individuals with a higher genetic risk, but not among those at a lower genetic risk [16]. Another cross-sectional study in 11,657 participants from a community-based population in China identified evidence that fruit intake alleviated the relationship between genetic predisposition and type 2 diabetes risk [39]. It has also been reported that dietary fiber intake may modify the association between genetic factors and type 2 diabetes incidence [40]. In the current study, we found that adherence to a low-inflammatory diet might significantly mitigate the risk of genetic factors on type 2 diabetes development.

The biological mechanisms responsible for the decreased type 2 diabetes risk attributable to a low-inflammatory diet, especially among participants at higher genetic risk, are multifactorial and incompletely understood. Consumption of pro-inflammatory foods, especially red meat, processed meat, and sweets which contain disease-promoting components such as saturated fat, advanced glycation end products, heme iron, nitrosamine, sodium nitrite, and nitroso compounds, may have toxic effects on pancreatic *β*-cells or impair insulin sensitivity [6]. Instead, adhering to a low-inflammatory diet may improve long-term hyperglycemia, metabolic disturbances, lipid profile, body composition, blood pressure, insulin sensitivity, and β-cell function [16, 35], which all play important roles in the development of type 2 diabetes. While genetic variants such as  TBC1D4 and TCF7L2 result in up to 50% increased risk of type 2 diabetes by diminishing the incretin effect and impairing glucagon-like peptide 1–induced insulin secretion, a fiber-rich diet may stimulate glucagon-like peptide 1 and mitigate this genetic risk [38, 40]. More experimental research is needed to provide biological insight into potential gene-diet interactions involved with type 2 diabetes risk.

The major strength of the present study is the large, well-characterized, population-based, prospective cohort with available genetic information. This sample provides a unique opportunity to detect associations between a low-inflammatory diet and type 2 diabetes incidence and whether these associations differ by genetic risk, while also controlling for potential confounders such as socioeconomic characteristics and lifestyle factors. Nonetheless, the limitations in the current study need to be acknowledged. First, as the information of fasting blood glucose were missing in the ascertain of incident diabetes in the UK Biobank, participants with undiagnosed type 2 diabetes might have been misclassified as type 2 diabetes-free, which could have caused an underestimation of the observed associations. Second, inflammatory diets in previous studies were developed based on a valid food frequency questionnaire (FFQ) which included questions about commonly consumed food items over a specified period (such as 12 months). Although dietary assessment in the present study was not based on an FFQ in the UK Biobank (only 29 questions about the consumption frequency of six food groups), the 24-h dietary assessment is more comprehensive and accurate and less likely to cause recall bias. Third, we did not exclude participants (45.6%) who had only one 24-h dietary assessment due to loss of sample size, which may less accurately reflect usual dietary habits [41]. However, we repeated analyses after excluding participants with only one 24-h dietary assessment, and the results were not meaningfully altered. Fourth, data on dietary patterns were obtained only at baseline. Any variation in dietary habits throughout follow-up were not captured, which could introduce bias. Fifth, data on other inflammatory markers such as TNF-α and IL-6 were not available in the UK Biobank, so we generated the IDI using only hsCRP levels. Sixth, the generalizability of our findings could be limited to the source population because of varied dietary habits in different populations. Finally, the participants were volunteers of entirely white British ancestry, so caution is required in generalizing our findings to individuals of other ethnic backgrounds.

## Conclusions

In conclusion, our study provides evidence that a low-inflammatory diet was associated with a decreased risk of type 2 diabetes and could delay type 2 diabetes onset among participants with normal blood glucose or prediabetes. Moreover, a low-inflammatory diet may mitigate genetic risk for diabetes. Our findings provide evidence that adhering to a low-inflammatory diet may support the prevention of type 2 diabetes.

### Supplementary Information

**Additional file 1: Method S1. **Genetic risk score.** Method S2. **Inflammatory diet index score.** Table S1. **Single nucleotide polymorphisms used to build the genetic risk score for type 2 diabetes. **Table S2. **Examples of food items constituting the 39 food groups used to calculate the inflammatory diet index from the Oxford WebQ questionnaire of the UK Biobank. **Table S3. **Factor loadings of reduced rank regression dietary pattern. **Table S4.** Spearman correlation coefficients between individual food groups with high-sensitivity C-reactive protein concentrations. **Table S5. **Relative concentrations of plasma high-sensitivity C-reactive protein (hsCRP) across tertiles of the inflammatory diet index in test and retest groups. **Table S6. **Examples of food items from the Oxford WebQ included in the 18 food groups to calculate empirical dietary inflammatory pattern scores. **Table S7. **Basic-adjusted hazard ratios and 50th percentile differences of incident type 2 diabetes in relation to a low-inflammatory diet. ** Table S8. **Hazard ratios for the association between levels of genetic risk score and type 2 diabetes. **Table S9. **Hazard ratios of type 2 diabetes according to joint categories of the inflammatory diet index and genetic risk score.**  Table S10. **Hazard ratios for the association between low-inflammatory diet and type 2 diabetes stratified by genetic risk score. **Table S11.** Additive interaction between genetic risk score and inflammatory diet index for type 2 diabetes. **Table S12. **Spearman correlation coefficients between individual food groups and high-sensitivity C-reactive protein concentrations among participants with at least two 24-h dietary assessments. **Table S13. **Hazard ratios and 50th percentile differences of incident type 2 diabetes in relation to empirical dietary inflammatory pattern score.** Table S14. **Hazard ratios and 50th percentile differences of incident type 2 diabetes in relation to a low-inflammatory diet in normoglycemia and prediabetes groups after excluding missing values for covariates.** Table S15. **Hazard ratios for the association between low-inflammatory diet and type 2 diabetes stratified by sex.** Table S16. **Hazard ratios for the association between low-inflammatory diet and type 2 diabetes stratified by age. **Table S17.** Hazard ratios for the association between low-inflammatory diet and type 2 diabetes stratified by physical activity. **Table S18. **Mediating effects of high-sensitivity C-reactive protein in the association between inflammatory diet index and incident type 2 diabetes. **Figure S1. **Mediating effects of high-sensitivity C-reactive protein in the association between inflammatory diet index and incident type 2 diabetes.

## Data Availability

The dataset supporting the conclusions of this article is available upon request from the UK Biobank. This study has been conducted using the UK Biobank Resource under project number 45676. Data from UK Biobank is not publicly available, and researchers can apply to use the data based on reasonable grounds at http://ukbiobank.ac.uk/register- apply/. The study protocols are published online at https://www.ukbiobank. ac.uk/learn-more-about-uk-biobank/about-us.

## Abbreviations

AP: Attributable proportion
BMI: Body mass index
CI: Confidence interval
DII: Dietary Inflammatory Index
EDIP: Empirical Dietary Inflammatory Pattern
FFQ: Food frequency questionnaire
GRS: Genetic risk score
hsCRP: High-sensitivity C-reactive protein
IDI: Inflammatory diet index
MPA: Moderate physical activity
OR: Odds ratio
RERI: Relative excess risk due to interaction
RRR: Reduced rank regression
SI: Synergy index
SNP: Single-nucleotide polymorphism
VPA: Vigorous physical activity
