# Supplementary material for: A low-inflammatory diet is associated with a lower incidence of diabetes: role of diabetes-related genetic risk
Source: BMC Med. 2023 Dec 5;21:483. doi: 10.1186/s12916-023-03190-1 (PMC10696657; doi:10.1186/s12916-023-03190-1)
Supplement: Supplementary file 1 — Additional file 1: Method S1. Genetic risk score. Method S2. Inflammatory diet index score. Table S1. Single nucleotide polymorphisms used to build the genetic risk score for type 2 diabetes. Table S2. Examples of food items constituting the 39 food groups used to calculate the inflammatory diet index from the Oxford WebQ questionnaire of the UK Biobank. Table S3. Factor loadings of reduced rank regression dietary pattern. Table S4. Spearman correlation coefficients between individual food groups with high-sensitivity C-reactive protein concentrations. Table S5. Relative concentrations of plasma high-sensitivity C-reactive protein (hsCRP) across tertiles of the inflammatory diet index in test and retest groups. Table S6. Examples of food items from the Oxford WebQ included in the 18 food groups to calculate empirical dietary inflammatory pattern scores. Table S7. Basic-adjusted hazard ratios and 50th percentile differences of incident type 2 diabetes in relation to a low-inflammatory diet. Table S8. Hazard ratios for the association between levels of genetic risk score and type 2 diabetes. Table S9. Hazard ratios of type 2 diabetes according to joint categories of the inflammatory diet index and genetic risk score. Table S10. Hazard ratios for the association between low-inflammatory diet and type 2 diabetes stratified by genetic risk score. Table S11. Additive interaction between genetic risk score and inflammatory diet index for type 2 diabetes. Table S12. Spearman correlation coefficients between individual food groups and high-sensitivity C-reactive protein concentrations among participants with at least two 24-h dietary assessments. Table S13. Hazard ratios and 50th percentile differences of incident type 2 diabetes in relation to empirical dietary inflammatory pattern score. Table S14. Hazard ratios and 50th percentile differences of incident type 2 diabetes in relation to a low-inflammatory diet in normoglycemia and prediabetes groups after excluding missing [file 12916_2023_3190_MOESM1_ESM.docx]

**Additional File 1:**

**Method S1.** Genetic risk score.

**Method S2.** Inflammatory diet index score.

**Table S1.** Single nucleotide polymorphisms (SNPs) used to build the genetic risk score for type 2 diabetes.

**Table S2.** Examples of food items constituting the 39 food groups used to calculate the inflammatory diet index (IDI) from the Oxford WebQ questionnaire of the UK Biobank.

**Table S3.** Factor loadings of reduced rank regression (RRR) dietary pattern.

**Table S4.** Food groups included in the inflammatory diet index (IDI) and Spearman correlation coefficients between individual food groups with high-sensitivity C-reactive protein (hsCRP) concentrations.

**Table** **S5.** Relative concentrations of plasma high-sensitivity C-reactive protein (hsCRP) across tertiles of the inflammatory diet index (IDI) in test and retest groups.

**Table S6.** Examples of food items from the Oxford WebQ included in the 18 food groups to calculate empirical dietary inflammatory pattern (EDIP) scores.

**Table S7.** Basic-adjusted hazard ratios (HRs), 50th percentile differences (PDs, years), and 95% confidence intervals (95% CIs) of incident type 2 diabetes (T2D) in relation to a low-inflammatory diet in normoglycemia and prediabetes groups.

**Table S8.** Hazard ratios (HRs) and 95% confidence intervals (95% CIs) for the association between levels of genetic risk score and type 2 diabetes (T2D) in normoglycemia and prediabetes groups.

**Table S9.** Hazard ratios (HRs) and 95% confidence intervals (95% CIs) of type 2 diabetes (T2D) according to joint categories of the inflammatory diet index (IDI) and genetic risk score in normoglycemia and prediabetes groups.

**Table** **S10.** Hazard ratios (HRs) and 95% confidence intervals (95% CIs) for the association between low-inflammatory diet and type 2 diabetes (T2D) in normoglycemia and prediabetes groups, stratified by genetic risk score.

**Table** **S11.** Additive interaction between genetic risk score (GRS) and inflammatory diet index (IDI) for type 2 diabetes (T2D) in normoglycemia and prediabetes groups.

**Table S12.** Food groups included in the inflammatory diet index (IDI) and Spearman correlation coefficients between individual food groups and high-sensitivity C-reactive protein (hsCRP) concentrations among participants with at least two 24-h dietary assessments.

**Table S13.** Hazard ratios (HRs), 50th percentile differences (PDs, years), and 95% confidence intervals (95% CIs) of incident type 2 diabetes (T2D) in relation to empirical dietary inflammatory pattern (EDIP) score in normoglycemia and prediabetes groups.

**Table S14.** Hazard ratios (HRs), 50th percentile differences (PDs, years), and 95% confidence intervals (95% CIs) of incident type 2 diabetes (T2D) in relation to a low-inflammatory diet in normoglycemia and prediabetes groups after excluding missing values for covariates.

**Table** **S15.** Hazard ratios (HRs) and 95% confidence intervals (95% CIs) for the association between low-inflammatory diet and type 2 diabetes (T2D) in normoglycemia and prediabetes groups, stratified by sex.

**Table** **S16.** Hazard ratios (HRs) and 95% confidence intervals (95% CIs) for the association between low-inflammatory diet and type 2 diabetes (T2D) in normoglycemia and prediabetes groups, stratified by age.

**Table** **S17.** Hazard ratios (HRs) and 95% confidence intervals (95% CIs) for the association between low-inflammatory diet and type 2 diabetes (T2D) in normoglycemia and prediabetes groups, stratified by physical activity.

**Table S18.** Mediating effects of high-sensitivity C-reactive protein (hsCRP) in the association between inflammatory diet index (IDI) and incident type 2 diabetes.

**Figure S1.** Mediating effects of high-sensitivity C-reactive protein (hsCRP) in the association between inflammatory diet index (IDI) and incident type 2 diabetes (T2D).

**Method S1.** Genetic risk score.

To generate a genetic risk score (GRS), each single nucleotide polymorphism (SNP) was recoded as 0, 1, or 2 according to the number of risk-increasing alleles. The score was calculated using the equation:

Score=β_1_×SNP_1_+β_2_×SNP_2_+…+β_n-1_×SNP_n-1_ +β_n_×SNP_n_

Where n is the total number of SNPs and β is the per-allele log odds ratio (OR) associated with SNP_n_. The effect size estimates of β were taken from a genome-wide association study carried out in subjects from Global Urate Genetics Consortium, a study of individuals of European ancestry. GRS was categorized as low (tertile 1), moderate (tertile 2), and high (tertile 3).

**Method S2.** Inflammatory diet index score.

The individual’s inflammatory diet index score was calculated by weighting the sum of the intake of the filtered food groups based on the regression coefficients derived from the final stepwise linear regression:

Score=0.00235×Butter+0.00204×Organ meat+0.00203×Other alcohol+0.002×Processed meat+0.00131×Red meat+0.00093815×Other meat+ 0.00068981×Ice-cream + 0.00064264×Poultry+ 0.00036889×Chocolate drink+0.00035341×Low calorie drink+0.00031821×Milk+0.00031512×Egg+0.00031063×Potato+0.00027962×Snack+0.00027053×Sweets+0.00024219×High calorie drink+0.00014106×Smoothie+0.00002603×Beer -0.00301×Nut-0.00082928×Vegetarian protein alternative -0.00078412×Starch -0.00076784×Breakfast cereal -0.00067483×Cheese -0.00036392×Dessert -0.00031898×Fish -0.00026867×Wine-0.0002396×Bread-0.0001813×Fruit-0.00017012×Pastry-0.0001197×Vegetable-0.00007641×Soup-0.00003361×Tea-0.00003295×Juice-0.00001992×Coffee

**Table S1.** Single nucleotide polymorphisms (SNPs) used to build the genetic risk score for type 2 diabetes.

| **SNPs** | **Chr.** | **Position** | **Closest gene** | **Other** | **EAF** | **Beta** |
| --- | --- | --- | --- | --- | --- | --- |
| rs10097617 | 8 | chr8:95961626 | T | C | 0.469 | 0.037 |
| rs10137475 | 14 | chr14:58797953 | G | A | 0.422 | 0.026 |
| rs10145154 | 14 | chr14:79939525 | T | C | 0.222 | 0.055 |
| rs10188334 | 2 | chr2:653874 | C | T | 0.828 | 0.05 |
| rs10240790 | 7 | chr7:89880949 | G | A | 0.714 | 0.028 |
| rs10305420 | 6 | chr6:39016636 | C | T | 0.608 | 0.032 |
| rs10404726 | 19 | chr19:18834514 | C | T | 0.533 | 0.028 |
| rs10406327 | 19 | chr19:33890838 | C | G | 0.521 | 0.037 |
| rs10407429 | 19 | chr19:46157237 | G | A | 0.574 | 0.054 |
| rs10471048 | 4 | chr4:83587562 | G | C | 0.337 | 0.034 |
| rs10490871 | 3 | chr3:35667761 | G | A | 0.365 | 0.027 |
| rs1059592 | 8 | chr8:22477778 | A | G | 0.354 | 0.027 |
| rs10737818 | 1 | chr1:235542023 | G | A | 0.643 | 0.04 |
| rs10750397 | 11 | chr11:128234144 | A | G | 0.278 | 0.048 |
| rs10769936 | 11 | chr11:8654528 | C | T | 0.725 | 0.035 |
| rs10771372 | 12 | chr12:27962260 | C | T | 0.803 | 0.072 |
| rs10771813 | 12 | chr12:31367856 | C | A | 0.554 | 0.026 |
| rs10787287 | 10 | chr10:112647195 | T | C | 0.767 | 0.036 |
| rs10788575 | 10 | chr10:89768584 | A | G | 0.164 | 0.035 |
| rs10811661 | 9 | chr9:22134094 | T | C | 0.826 | 0.138 |
| rs10821311 | 9 | chr9:96943059 | A | G | 0.318 | 0.036 |
| rs10830963 | 11 | chr11:92708710 | G | C | 0.274 | 0.089 |
| rs10841868 | 12 | chr12:21781246 | G | T | 0.74 | 0.032 |
| rs10844518 | 12 | chr12:33410780 | G | A | 0.279 | 0.033 |
| rs10882891 | 10 | chr10:99059645 | C | A | 0.403 | 0.031 |
| rs10889560 | 1 | chr1:65989878 | A | C | 0.082 | 0.047 |
| rs10899283 | 11 | chr11:76505202 | C | T | 0.778 | 0.031 |
| rs10915188 | 1 | chr1:29024956 | A | G | 0.583 | 0.029 |
| rs10916784 | 1 | chr1:20729451 | G | C | 0.581 | 0.027 |
| rs10937208 | 3 | chr3:184877626 | G | A | 0.136 | 0.044 |
| rs10937721 | 4 | chr4:6306763 | C | G | 0.591 | 0.084 |
| rs10938398 | 4 | chr4:45186139 | A | G | 0.434 | 0.043 |
| rs10963942 | 9 | chr9:19080352 | G | A | 0.39 | 0.037 |
| rs10974438 | 9 | chr9:4291928 | C | A | 0.351 | 0.047 |
| rs10998304 | 10 | chr10:70342775 | C | T | 0.451 | 0.031 |
| rs11038672 | 11 | chr11:45846498 | C | G | 0.475 | 0.029 |
| rs11048458 | 12 | chr12:26465585 | T | C | 0.243 | 0.046 |
| rs11073147 | 15 | chr15:36392562 | G | A | 0.551 | 0.025 |
| rs11078916 | 17 | chr17:37746307 | T | C | 0.276 | 0.037 |
| rs11108094 | 12 | chr12:95928113 | A | C | 0.069 | 0.06 |
| rs11117364 | 16 | chr16:88132199 | G | A | 0.676 | 0.029 |
| rs1111875 | 10 | chr10:94462882 | C | T | 0.591 | 0.092 |
| rs11129735 | 3 | chr3:36870230 | A | G | 0.455 | 0.026 |
| rs11155073 | 6 | chr6:139837128 | T | C | 0.409 | 0.03 |
| rs11181613 | 12 | chr12:43046449 | C | A | 0.849 | 0.043 |
| rs111824905 | 1 | chr1:28110797 | C | T | 0.052 | 0.069 |
| rs11201999 | 10 | chr10:88124501 | C | T | 0.541 | 0.026 |
| rs1122518 | 7 | chr7:13900325 | C | T | 0.476 | 0.026 |
| rs11257655 | 10 | chr10:12307894 | T | C | 0.207 | 0.091 |
| rs112667817 | 5 | chr5:137823156 | C | T | 0.883 | 0.061 |
| rs1127215 | 1 | chr1:117532790 | C | T | 0.58 | 0.043 |
| rs113036477 | 12 | chr12:97848227 | C | T | 0.943 | 0.072 |
| rs114136102 | 5 | chr5:36084426 | C | T | 0.04 | 0.072 |
| rs114447556 | 4 | chr4:53207093 | T | C | 0.082 | 0.058 |
| rs11558471 | 8 | chr8:118185733 | A | G | 0.682 | 0.103 |
| rs11602873 | 11 | chr11:72460762 | A | T | 0.843 | 0.098 |
| rs11614914 | 12 | chr12:133070294 | T | C | 0.327 | 0.039 |
| rs11616380 | 13 | chr13:80705315 | G | T | 0.712 | 0.079 |
| rs11639470 | 15 | chr15:39639171 | C | G | 0.535 | 0.027 |
| rs11646052 | 16 | chr16:85716463 | G | A | 0.394 | 0.026 |
| rs11655898 | 17 | chr17:62201374 | C | T | 0.07 | 0.059 |
| rs11657964 | 17 | chr17:36100767 | A | G | 0.397 | 0.059 |
| rs11662800 | 18 | chr18:13271367 | A | G | 0.415 | 0.028 |
| rs11666603 | 19 | chr19:12496934 | C | T | 0.764 | 0.033 |
| rs11667244 | 19 | chr19:47580185 | G | A | 0.715 | 0.035 |
| rs11680058 | 2 | chr2:16574669 | A | G | 0.87 | 0.056 |
| rs116861182 | 11 | chr11:55588216 | C | A | 0.057 | 0.064 |
| rs117001013 | 22 | chr22:32348841 | C | T | 0.914 | 0.047 |
| rs11708067 | 3 | chr3:123065778 | A | G | 0.757 | 0.078 |
| rs11716527 | 3 | chr3:89986280 | C | T | 0.105 | 0.049 |
| rs117173251 | 8 | chr8:4186731 | T | C | 0.033 | 0.077 |
| rs11723275 | 4 | chr4:77528821 | C | A | 0.464 | 0.027 |
| rs117233107 | 12 | chr12:4328521 | G | A | 0.985 | 0.325 |
| rs117316450 | 11 | chr11:14518419 | G | C | 0.021 | 0.131 |
| rs11759026 | 6 | chr6:126792095 | G | A | 0.228 | 0.065 |
| rs11793831 | 9 | chr9:23362311 | T | G | 0.415 | 0.027 |
| rs11830243 | 12 | chr12:132544694 | T | C | 0.108 | 0.044 |
| rs11870735 | 17 | chr17:481604 | T | C | 0.18 | 0.034 |
| rs11922794 | 3 | chr3:72813582 | C | G | 0.245 | 0.03 |
| rs11940813 | 4 | chr4:20210953 | G | A | 0.133 | 0.037 |
| rs1194606 | 1 | chr1:154294260 | C | T | 0.235 | 0.03 |
| rs11967262 | 6 | chr6:43760327 | G | C | 0.488 | 0.037 |
| rs12001437 | 9 | chr9:34074476 | C | T | 0.368 | 0.034 |
| rs12048743 | 1 | chr1:205114873 | G | C | 0.43 | 0.032 |
| rs12056338 | 8 | chr8:12643055 | T | G | 0.416 | 0.031 |
| rs12187734 | 5 | chr5:51763665 | C | T | 0.515 | 0.029 |
| rs12194820 | 6 | chr6:127401978 | A | T | 0.755 | 0.046 |
| rs1225052 | 3 | chr3:131644937 | G | A | 0.375 | 0.027 |
| rs12263348 | 10 | chr10:65305252 | T | C | 0.348 | 0.028 |
| rs12305809 | 12 | chr12:133777466 | G | A | 0.606 | 0.033 |
| rs12454712 | 18 | chr18:60845884 | T | C | 0.623 | 0.041 |
| rs12463719 | 2 | chr2:203450680 | A | G | 0.283 | 0.032 |
| rs12505942 | 4 | chr4:140906390 | T | C | 0.656 | 0.03 |
| rs12509379 | 4 | chr4:129179458 | T | G | 0.202 | 0.031 |
| rs12519500 | 5 | chr5:78436905 | C | A | 0.65 | 0.038 |
| rs12539264 | 7 | chr7:48839003 | G | A | 0.283 | 0.029 |
| rs12602834 | 17 | chr17:29637308 | G | A | 0.385 | 0.029 |
| rs1260326 | 2 | chr2:27730940 | C | T | 0.604 | 0.064 |
| rs12625671 | 20 | chr20:42994812 | C | T | 0.107 | 0.065 |
| rs12741141 | 1 | chr1:6669970 | G | C | 0.355 | 0.04 |
| rs12773019 | 10 | chr10:73835274 | G | C | 0.027 | 0.089 |
| rs12820906 | 12 | chr12:123493123 | A | G | 0.753 | 0.043 |
| rs12823740 | 12 | chr12:124458002 | C | A | 0.666 | 0.041 |
| rs12890750 | 14 | chr14:103860309 | G | T | 0.651 | 0.028 |
| rs12910361 | 15 | chr15:77782335 | G | A | 0.712 | 0.072 |
| rs12912777 | 15 | chr15:38852386 | T | C | 0.125 | 0.059 |
| rs12917449 | 15 | chr15:74331659 | C | A | 0.196 | 0.036 |
| rs12920022 | 16 | chr16:89564055 | A | T | 0.161 | 0.039 |
| rs12933120 | 16 | chr16:3634746 | A | C | 0.143 | 0.042 |
| rs12977104 | 19 | chr19:4949921 | A | G | 0.204 | 0.041 |
| rs12992995 | 2 | chr2:175197545 | C | A | 0.724 | 0.031 |
| rs13005841 | 2 | chr2:212302573 | A | T | 0.708 | 0.029 |
| rs13020443 | 2 | chr2:152167830 | C | T | 0.508 | 0.031 |
| rs13155752 | 5 | chr5:44680687 | C | A | 0.396 | 0.032 |
| rs13237518 | 7 | chr7:12269593 | A | C | 0.415 | 0.029 |
| rs13262861 | 8 | chr8:41508577 | C | A | 0.825 | 0.102 |
| rs133015 | 22 | chr22:38572526 | C | G | 0.56 | 0.029 |
| rs13389219 | 2 | chr2:165528876 | C | T | 0.606 | 0.065 |
| rs13414140 | 2 | chr2:43671176 | C | T | 0.885 | 0.118 |
| rs1412234 | 9 | chr9:28410683 | C | T | 0.327 | 0.044 |
| rs1421085 | 16 | chr16:53800954 | C | T | 0.403 | 0.118 |
| rs1430780 | 2 | chr2:67878328 | T | C | 0.32 | 0.027 |
| rs1431819 | 9 | chr9:116943357 | G | A | 0.696 | 0.029 |
| rs1437055 | 3 | chr3:86831077 | A | C | 0.617 | 0.027 |
| rs144245804 | 11 | chr11:69453044 | G | A | 0.974 | 0.13 |
| rs1449348 | 3 | chr3:168225055 | C | T | 0.86 | 0.045 |
| rs1451506 | 17 | chr17:57407019 | A | G | 0.115 | 0.042 |
| rs145904381 | 1 | chr1:151017991 | T | C | 0.988 | 0.174 |
| rs146886108 | 5 | chr5:14751305 | C | T | 0.993 | 0.39 |
| rs1475655 | 13 | chr13:91963080 | A | T | 0.739 | 0.044 |
| rs149364428 | 8 | chr8:97737741 | A | G | 0.008 | 0.224 |
| rs1493694 | 1 | chr1:120526982 | T | C | 0.107 | 0.071 |
| rs1513272 | 7 | chr7:28200097 | C | T | 0.498 | 0.081 |
| rs1517037 | 18 | chr18:56878274 | C | T | 0.812 | 0.038 |
| rs152839 | 5 | chr5:50145266 | C | T | 0.584 | 0.026 |
| rs1531583 | 4 | chr4:744972 | T | G | 0.039 | 0.099 |
| rs1561927 | 8 | chr8:129568078 | C | T | 0.268 | 0.035 |
| rs1562398 | 7 | chr7:130457931 | G | C | 0.415 | 0.041 |
| rs1573090 | 6 | chr6:137302159 | T | G | 0.538 | 0.045 |
| rs1656794 | 17 | chr17:75386909 | G | A | 0.725 | 0.031 |
| rs17035289 | 4 | chr4:106048291 | C | T | 0.16 | 0.043 |
| rs17036160 | 3 | chr3:12329783 | C | T | 0.882 | 0.103 |
| rs1705263 | 12 | chr12:71523043 | C | A | 0.56 | 0.04 |
| rs17091891 | 8 | chr8:19843171 | T | C | 0.88 | 0.042 |
| rs17175860 | 19 | chr19:7235146 | G | A | 0.194 | 0.045 |
| rs1724557 | 4 | chr4:137094048 | C | A | 0.413 | 0.025 |
| rs17265513 | 20 | chr20:39832628 | C | T | 0.199 | 0.033 |
| rs17294565 | 8 | chr8:14124809 | C | A | 0.384 | 0.027 |
| rs17354348 | 2 | chr2:213835977 | A | G | 0.743 | 0.029 |
| rs17439448 | 7 | chr7:40816653 | T | C | 0.122 | 0.04 |
| rs174541 | 11 | chr11:61565908 | T | C | 0.64 | 0.029 |
| rs1752169 | 9 | chr9:126586563 | A | C | 0.25 | 0.032 |
| rs17522122 | 14 | chr14:33302882 | T | G | 0.47 | 0.034 |
| rs17624303 | 2 | chr2:105148418 | C | T | 0.73 | 0.029 |
| rs17684074 | 18 | chr18:54675384 | G | C | 0.748 | 0.031 |
| rs177045 | 10 | chr10:71321279 | G | A | 0.315 | 0.039 |
| rs17772814 | 8 | chr8:128711742 | G | A | 0.918 | 0.075 |
| rs17791513 | 9 | chr9:81905590 | A | G | 0.938 | 0.069 |
| rs17818197 | 8 | chr8:25872634 | G | A | 0.224 | 0.035 |
| rs1783541 | 11 | chr11:65294799 | T | C | 0.217 | 0.048 |
| rs1815591 | 20 | chr20:61277014 | A | T | 0.389 | 0.034 |
| rs181752889 | 3 | chr3:128579324 | T | C | 0.002 | 0.282 |
| rs1819564 | 6 | chr6:51505337 | A | T | 0.028 | 0.076 |
| rs1872635 | 12 | chr12:54541750 | A | G | 0.688 | 0.028 |
| rs1874832 | 15 | chr15:67260238 | G | A | 0.154 | 0.038 |
| rs1929883 | 9 | chr9:81344701 | G | A | 0.584 | 0.039 |
| rs1968204 | 7 | chr7:102800137 | T | C | 0.093 | 0.057 |
| rs197379 | 1 | chr1:112292303 | C | T | 0.38 | 0.027 |
| rs197482 | 6 | chr6:143069315 | C | T | 0.623 | 0.03 |
| rs2008027 | 6 | chr6:126052359 | G | A | 0.516 | 0.027 |
| rs2011603 | 4 | chr4:18025484 | A | G | 0.737 | 0.038 |
| rs2032912 | 16 | chr16:69568303 | G | T | 0.59 | 0.042 |
| rs2033159 | 2 | chr2:145261174 | C | A | 0.229 | 0.035 |
| rs2055997 | 4 | chr4:76535086 | G | A | 0.708 | 0.031 |
| rs2056857 | 14 | chr14:77300863 | C | T | 0.582 | 0.026 |
| rs2080090 | 17 | chr17:65828371 | A | T | 0.189 | 0.053 |
| rs2103132 | 7 | chr7:69782073 | C | G | 0.247 | 0.032 |
| rs2115107 | 19 | chr19:7968168 | A | G | 0.382 | 0.038 |
| rs2191349 | 7 | chr7:15064309 | T | G | 0.548 | 0.066 |
| rs2237895 | 11 | chr11:2857194 | C | A | 0.415 | 0.073 |
| rs2243102 | 17 | chr17:4839149 | C | T | 0.406 | 0.026 |
| rs2250301 | 10 | chr10:104548393 | G | A | 0.751 | 0.032 |
| rs2252221 | 20 | chr20:51621922 | G | A | 0.526 | 0.025 |
| rs2268078 | 20 | chr20:32596704 | A | G | 0.641 | 0.039 |
| rs2269247 | 1 | chr1:64107284 | C | T | 0.818 | 0.035 |
| rs2280141 | 10 | chr10:124193181 | T | G | 0.528 | 0.045 |
| rs2289739 | 15 | chr15:41801512 | T | G | 0.345 | 0.05 |
| rs2290203 | 15 | chr15:91512067 | A | G | 0.198 | 0.056 |
| rs2292662 | 3 | chr3:63897215 | C | T | 0.849 | 0.056 |
| rs2297508 | 17 | chr17:17715317 | C | G | 0.351 | 0.033 |
| rs2313211 | 3 | chr3:183738626 | T | A | 0.445 | 0.029 |
| rs2336938 | 1 | chr1:206618799 | A | C | 0.477 | 0.03 |
| rs2403221 | 11 | chr11:9852475 | A | G | 0.674 | 0.032 |
| rs2409742 | 8 | chr8:11069960 | C | T | 0.512 | 0.036 |
| rs2410767 | 5 | chr5:87705268 | C | G | 0.787 | 0.033 |
| rs2426439 | 20 | chr20:50999627 | C | T | 0.633 | 0.037 |
| rs243018 | 2 | chr2:60586707 | G | C | 0.451 | 0.056 |
| rs2435907 | 15 | chr15:57333416 | A | G | 0.582 | 0.029 |
| rs253412 | 5 | chr5:74955841 | A | G | 0.662 | 0.046 |
| rs2540949 | 2 | chr2:65284231 | A | T | 0.624 | 0.05 |
| rs256904 | 5 | chr5:55810305 | T | A | 0.746 | 0.069 |
| rs2581787 | 3 | chr3:53127677 | T | G | 0.56 | 0.025 |
| rs2583921 | 12 | chr12:66170481 | C | A | 0.089 | 0.095 |
| rs2613503 | 1 | chr1:72839774 | A | C | 0.803 | 0.039 |
| rs2658746 | 18 | chr18:74582340 | C | T | 0.378 | 0.03 |
| rs2675662 | 10 | chr10:75599127 | A | G | 0.563 | 0.027 |
| rs2725371 | 8 | chr8:30854033 | A | G | 0.303 | 0.037 |
| rs2732480 | 12 | chr12:48736303 | C | A | 0.572 | 0.034 |
| rs2733289 | 12 | chr12:41838235 | C | T | 0.478 | 0.03 |
| rs2737226 | 8 | chr8:116639474 | T | C | 0.392 | 0.038 |
| rs2796441 | 9 | chr9:84308948 | G | A | 0.581 | 0.059 |
| rs2820444 | 1 | chr1:219741820 | G | A | 0.702 | 0.047 |
| rs28429551 | 9 | chr9:139243334 | A | T | 0.755 | 0.073 |
| rs2862954 | 10 | chr10:101912064 | T | C | 0.502 | 0.029 |
| rs2876826 | 7 | chr7:50581972 | G | A | 0.223 | 0.031 |
| rs28819812 | 4 | chr4:157652753 | C | A | 0.678 | 0.038 |
| rs2908286 | 7 | chr7:44234737 | T | C | 0.178 | 0.068 |
| rs2925979 | 16 | chr16:81534790 | T | C | 0.299 | 0.045 |
| rs2933211 | 14 | chr14:47313541 | A | G | 0.493 | 0.027 |
| rs2972145 | 2 | chr2:227101309 | C | T | 0.646 | 0.09 |
| rs3020781 | 1 | chr1:155269776 | G | A | 0.265 | 0.033 |
| rs303760 | 18 | chr18:21083738 | T | C | 0.345 | 0.034 |
| rs3094682 | 6 | chr6:31264461 | C | A | 0.815 | 0.06 |
| rs3111316 | 19 | chr19:13038415 | A | G | 0.587 | 0.044 |
| rs314879 | 13 | chr13:23309382 | C | T | 0.212 | 0.039 |
| rs3176466 | 1 | chr1:51438365 | C | T | 0.91 | 0.064 |
| rs329122 | 5 | chr5:133864599 | A | G | 0.421 | 0.026 |
| rs340874 | 1 | chr1:214159256 | C | T | 0.568 | 0.067 |
| rs34143602 | 15 | chr15:63940058 | G | A | 0.41 | 0.035 |
| rs34298980 | 6 | chr6:40409243 | T | C | 0.491 | 0.038 |
| rs34329895 | 2 | chr2:208870017 | A | G | 0.396 | 0.028 |
| rs34340810 | 8 | chr8:105661926 | G | C | 0.927 | 0.054 |
| rs34506349 | 2 | chr2:100598726 | G | A | 0.959 | 0.068 |
| rs34573045 | 3 | chr3:149196752 | G | C | 0.433 | 0.031 |
| rs34584161 | 13 | chr13:26776999 | A | G | 0.764 | 0.052 |
| rs34589210 | 2 | chr2:112795492 | A | G | 0.145 | 0.039 |
| rs346240 | 18 | chr18:40063830 | G | A | 0.204 | 0.031 |
| rs348330 | 1 | chr1:229672955 | G | A | 0.367 | 0.053 |
| rs34845373 | 2 | chr2:25635771 | A | G | 0.727 | 0.037 |
| rs34965774 | 12 | chr12:118412373 | A | G | 0.13 | 0.052 |
| rs34990153 | 8 | chr8:9996389 | A | G | 0.556 | 0.038 |
| rs35004890 | 19 | chr19:1224286 | T | G | 0.231 | 0.036 |
| rs35169799 | 11 | chr11:64031241 | T | C | 0.063 | 0.05 |
| rs35352848 | 3 | chr3:23455582 | T | C | 0.796 | 0.062 |
| rs35895680 | 17 | chr17:47060322 | C | A | 0.673 | 0.056 |
| rs35901985 | 4 | chr4:186580062 | A | G | 0.821 | 0.035 |
| rs36051838 | 10 | chr10:34018730 | C | T | 0.086 | 0.044 |
| rs36111056 | 15 | chr15:83461873 | G | A | 0.789 | 0.034 |
| rs362307 | 4 | chr4:3241845 | T | C | 0.074 | 0.05 |
| rs3742305 | 13 | chr13:31036642 | C | G | 0.732 | 0.03 |
| rs3747207 | 22 | chr22:44324855 | A | G | 0.215 | 0.047 |
| rs3755879 | 4 | chr4:96114385 | A | G | 0.301 | 0.033 |
| rs3757969 | 8 | chr8:145551199 | G | C | 0.374 | 0.048 |
| rs3764002 | 12 | chr12:108618630 | C | T | 0.738 | 0.04 |
| rs3798519 | 6 | chr6:50788778 | C | A | 0.179 | 0.05 |
| rs38221 | 7 | chr7:15926228 | T | C | 0.253 | 0.033 |
| rs3872707 | 3 | chr3:9514016 | A | G | 0.124 | 0.045 |
| rs3887925 | 3 | chr3:186665645 | T | C | 0.549 | 0.042 |
| rs39328 | 7 | chr7:103444978 | T | C | 0.424 | 0.028 |
| rs41276588 | 1 | chr1:118148384 | A | G | 0.285 | 0.038 |
| rs4132228 | 3 | chr3:64708114 | C | T | 0.711 | 0.047 |
| rs419842 | 20 | chr20:42310811 | T | A | 0.842 | 0.041 |
| rs429358 | 19 | chr19:45411941 | T | C | 0.846 | 0.073 |
| rs4325 | 17 | chr17:61563200 | C | A | 0.532 | 0.035 |
| rs4397977 | 13 | chr13:41688401 | A | G | 0.342 | 0.029 |
| rs4465929 | 3 | chr3:15741389 | T | C | 0.402 | 0.03 |
| rs448918 | 9 | chr9:136885979 | A | G | 0.265 | 0.033 |
| rs4655617 | 1 | chr1:67010654 | C | A | 0.44 | 0.028 |
| rs4688760 | 3 | chr3:49980596 | T | C | 0.689 | 0.034 |
| rs4709746 | 6 | chr6:164133001 | C | T | 0.865 | 0.058 |
| rs4714422 | 6 | chr6:41012405 | G | A | 0.245 | 0.029 |
| rs4721089 | 7 | chr7:1872921 | T | C | 0.782 | 0.034 |
| rs4734193 | 8 | chr8:110140564 | C | A | 0.527 | 0.034 |
| rs4776970 | 15 | chr15:68080886 | A | T | 0.642 | 0.029 |
| rs4796224 | 17 | chr17:34842521 | G | A | 0.472 | 0.025 |
| rs4805681 | 19 | chr19:31835516 | C | T | 0.605 | 0.027 |
| rs480840 | 11 | chr11:74625997 | C | T | 0.429 | 0.025 |
| rs4809369 | 20 | chr20:62470785 | G | A | 0.548 | 0.034 |
| rs4845987 | 1 | chr1:11306279 | C | G | 0.709 | 0.028 |
| rs484943 | 15 | chr15:40398754 | T | C | 0.338 | 0.033 |
| rs4865796 | 5 | chr5:53272664 | A | G | 0.692 | 0.047 |
| rs4899280 | 14 | chr14:69526307 | T | C | 0.332 | 0.028 |
| rs4916253 | 1 | chr1:172361032 | G | T | 0.433 | 0.027 |
| rs4929965 | 11 | chr11:2197286 | A | G | 0.379 | 0.062 |
| rs495203 | 9 | chr9:136145240 | T | C | 0.318 | 0.049 |
| rs4976033 | 5 | chr5:67714246 | G | A | 0.401 | 0.028 |
| rs4984980 | 16 | chr16:968292 | A | G | 0.182 | 0.035 |
| rs5219 | 11 | chr11:17409572 | T | C | 0.357 | 0.069 |
| rs534043 | 7 | chr7:100312724 | G | A | 0.887 | 0.045 |
| rs543159 | 6 | chr6:160776017 | C | A | 0.527 | 0.032 |
| rs545608 | 1 | chr1:177899121 | C | G | 0.206 | 0.036 |
| rs555784 | 9 | chr9:85318704 | T | A | 0.617 | 0.03 |
| rs55812705 | 6 | chr6:111738793 | T | C | 0.755 | 0.031 |
| rs55857387 | 16 | chr16:300388 | T | C | 0.801 | 0.052 |
| rs56337234 | 4 | chr4:1784403 | C | T | 0.506 | 0.041 |
| rs56348580 | 12 | chr12:121432117 | G | C | 0.692 | 0.058 |
| rs56394279 | 3 | chr3:160171092 | C | T | 0.471 | 0.031 |
| rs567185 | 1 | chr1:201763499 | T | C | 0.649 | 0.037 |
| rs5751061 | 22 | chr22:41593873 | G | T | 0.629 | 0.026 |
| rs5753043 | 22 | chr22:30588041 | C | A | 0.908 | 0.061 |
| rs576674 | 13 | chr13:33554302 | G | A | 0.167 | 0.061 |
| rs5771069 | 22 | chr22:50435480 | G | A | 0.497 | 0.033 |
| rs583769 | 7 | chr7:18331915 | A | G | 0.246 | 0.031 |
| rs58542926 | 19 | chr19:19379549 | T | C | 0.074 | 0.089 |
| rs60519666 | 6 | chr6:107427166 | G | A | 0.676 | 0.035 |
| rs6066138 | 20 | chr20:45594711 | G | A | 0.718 | 0.045 |
| rs61736066 | 17 | chr17:70645032 | G | A | 0.913 | 0.051 |
| rs61779275 | 1 | chr1:39820310 | T | C | 0.212 | 0.075 |
| rs62034975 | 16 | chr16:20392415 | C | G | 0.302 | 0.031 |
| rs62075585 | 17 | chr17:76762039 | G | A | 0.473 | 0.03 |
| rs62262091 | 3 | chr3:47693664 | T | C | 0.094 | 0.056 |
| rs62271373 | 3 | chr3:150066540 | A | T | 0.06 | 0.069 |
| rs62310934 | 4 | chr4:48880627 | C | G | 0.618 | 0.03 |
| rs62450857 | 7 | chr7:4683258 | A | G | 0.134 | 0.039 |
| rs62492368 | 7 | chr7:150537635 | A | G | 0.304 | 0.034 |
| rs62515938 | 8 | chr8:57483013 | T | C | 0.261 | 0.029 |
| rs62618693 | 11 | chr11:32956492 | C | T | 0.955 | 0.085 |
| rs6432613 | 2 | chr2:161145612 | G | A | 0.724 | 0.039 |
| rs6438247 | 3 | chr3:115084080 | C | T | 0.131 | 0.044 |
| rs6459733 | 7 | chr7:156930550 | G | C | 0.662 | 0.051 |
| rs6495182 | 15 | chr15:75814388 | C | T | 0.749 | 0.041 |
| rs663640 | 18 | chr18:57846077 | T | C | 0.219 | 0.05 |
| rs6685701 | 1 | chr1:26868639 | A | G | 0.271 | 0.03 |
| rs67013744 | 12 | chr12:6681786 | G | A | 0.166 | 0.035 |
| rs6715901 | 2 | chr2:179650954 | G | A | 0.503 | 0.027 |
| rs6741676 | 2 | chr2:181618654 | A | G | 0.662 | 0.032 |
| rs6766859 | 3 | chr3:138055136 | C | T | 0.372 | 0.033 |
| rs67755137 | 7 | chr7:74108135 | A | G | 0.193 | 0.033 |
| rs6777684 | 3 | chr3:187741842 | G | A | 0.61 | 0.057 |
| rs6819331 | 4 | chr4:153504295 | C | T | 0.681 | 0.04 |
| rs6821438 | 4 | chr4:95091911 | A | G | 0.53 | 0.029 |
| rs684214 | 17 | chr17:40696915 | T | C | 0.28 | 0.042 |
| rs686998 | 3 | chr3:173119768 | G | A | 0.53 | 0.027 |
| rs6878122 | 5 | chr5:76427311 | G | A | 0.319 | 0.055 |
| rs6976111 | 7 | chr7:117495667 | A | C | 0.302 | 0.032 |
| rs7026688 | 9 | chr9:125975397 | G | A | 0.863 | 0.044 |
| rs703981 | 10 | chr10:80942855 | G | C | 0.543 | 0.061 |
| rs7046845 | 9 | chr9:97804641 | A | C | 0.911 | 0.047 |
| rs7071943 | 10 | chr10:93956552 | G | T | 0.659 | 0.044 |
| rs7099048 | 10 | chr10:77647107 | A | G | 0.515 | 0.028 |
| rs7132908 | 12 | chr12:50263148 | A | G | 0.384 | 0.033 |
| rs7163757 | 15 | chr15:62391608 | C | T | 0.571 | 0.041 |
| rs7188071 | 16 | chr16:28917644 | T | C | 0.353 | 0.029 |
| rs7219033 | 17 | chr17:9787958 | A | G | 0.329 | 0.029 |
| rs7220340 | 17 | chr17:27566326 | A | G | 0.46 | 0.027 |
| rs7240767 | 18 | chr18:7070642 | C | T | 0.387 | 0.037 |
| rs72501964 | 4 | chr4:1267203 | G | T | 0.963 | 0.083 |
| rs72692805 | 1 | chr1:149894355 | G | A | 0.921 | 0.054 |
| rs72695645 | 4 | chr4:185713608 | G | A | 0.859 | 0.061 |
| rs727734 | 6 | chr6:15475051 | A | T | 0.761 | 0.03 |
| rs72802342 | 16 | chr16:75234872 | C | A | 0.923 | 0.115 |
| rs72803684 | 2 | chr2:26192802 | T | C | 0.047 | 0.069 |
| rs72836348 | 2 | chr2:111888043 | G | A | 0.898 | 0.056 |
| rs72926932 | 18 | chr18:53050646 | C | A | 0.08 | 0.075 |
| rs72951506 | 6 | chr6:118011723 | C | T | 0.844 | 0.042 |
| rs73347525 | 14 | chr14:101255172 | A | G | 0.818 | 0.049 |
| rs73872717 | 3 | chr3:141134569 | C | T | 0.954 | 0.086 |
| rs74672008 | 3 | chr3:152451616 | G | A | 0.964 | 0.08 |
| rs74804697 | 15 | chr15:52588722 | C | G | 0.955 | 0.084 |
| rs75332279 | 15 | chr15:53099306 | C | T | 0.1 | 0.056 |
| rs7538321 | 1 | chr1:205789455 | T | A | 0.127 | 0.042 |
| rs75401573 | 22 | chr22:29805444 | C | T | 0.922 | 0.051 |
| rs75432112 | 5 | chr5:102586407 | A | G | 0.05 | 0.134 |
| rs7558413 | 2 | chr2:18721662 | A | G | 0.58 | 0.029 |
| rs7559658 | 2 | chr2:147920213 | C | T | 0.189 | 0.034 |
| rs756145 | 9 | chr9:1039939 | A | G | 0.306 | 0.029 |
| rs7561798 | 2 | chr2:228973660 | G | A | 0.481 | 0.028 |
| rs7568172 | 2 | chr2:158335340 | G | A | 0.937 | 0.067 |
| rs75686861 | 4 | chr4:145621328 | A | G | 0.092 | 0.047 |
| rs75693095 | 7 | chr7:23440057 | C | G | 0.022 | 0.112 |
| rs75756987 | 21 | chr21:47767295 | G | C | 0.896 | 0.044 |
| rs7609422 | 2 | chr2:146348037 | G | A | 0.408 | 0.03 |
| rs7619708 | 3 | chr3:195810187 | T | C | 0.76 | 0.033 |
| rs76263492 | 3 | chr3:54828827 | T | G | 0.043 | 0.068 |
| rs7656001 | 4 | chr4:91243865 | A | G | 0.55 | 0.026 |
| rs7660000 | 4 | chr4:89751858 | C | T | 0.718 | 0.031 |
| rs7695096 | 4 | chr4:103932556 | C | T | 0.514 | 0.038 |
| rs7719891 | 5 | chr5:86577352 | G | A | 0.247 | 0.04 |
| rs7739842 | 6 | chr6:131954797 | G | T | 0.189 | 0.033 |
| rs7756992 | 6 | chr6:20679709 | G | A | 0.265 | 0.122 |
| rs7858727 | 9 | chr9:111936128 | C | A | 0.205 | 0.034 |
| rs7867635 | 9 | chr9:20241069 | C | T | 0.411 | 0.037 |
| rs7903146 | 10 | chr10:114758349 | T | C | 0.287 | 0.28 |
| rs79489938 | 1 | chr1:147121000 | G | A | 0.984 | 0.113 |
| rs798549 | 7 | chr7:2760750 | C | A | 0.269 | 0.03 |
| rs799661 | 14 | chr14:35390146 | C | T | 0.891 | 0.043 |
| rs8005994 | 14 | chr14:29744532 | A | G | 0.645 | 0.027 |
| rs8008540 | 14 | chr14:74948180 | C | T | 0.563 | 0.03 |
| rs8010382 | 14 | chr14:91963722 | G | A | 0.414 | 0.032 |
| rs8018512 | 14 | chr14:38818723 | G | A | 0.749 | 0.037 |
| rs8031576 | 15 | chr15:90380214 | C | A | 0.28 | 0.057 |
| rs8033609 | 15 | chr15:60938816 | A | C | 0.542 | 0.027 |
| rs8054556 | 16 | chr16:29958216 | A | G | 0.467 | 0.036 |
| rs8071043 | 17 | chr17:3988451 | C | T | 0.33 | 0.054 |
| rs827237 | 10 | chr10:72648336 | T | C | 0.199 | 0.037 |
| rs838735 | 2 | chr2:234324192 | C | G | 0.385 | 0.029 |
| rs844215 | 3 | chr3:71656045 | C | T | 0.583 | 0.026 |
| rs858519 | 17 | chr17:7531965 | T | C | 0.443 | 0.026 |
| rs867489 | 20 | chr20:48833957 | C | T | 0.536 | 0.031 |
| rs878017 | 10 | chr10:13566204 | A | G | 0.527 | 0.035 |
| rs890940 | 5 | chr5:158026744 | T | C | 0.211 | 0.048 |
| rs911300 | 20 | chr20:57387262 | G | A | 0.543 | 0.035 |
| rs917195 | 7 | chr7:30728452 | C | T | 0.768 | 0.047 |
| rs9275184 | 6 | chr6:32654714 | C | T | 0.103 | 0.098 |
| rs9296095 | 6 | chr6:33542523 | T | C | 0.821 | 0.034 |
| rs9316500 | 13 | chr13:51094114 | T | G | 0.704 | 0.047 |
| rs9319382 | 13 | chr13:28245127 | C | T | 0.685 | 0.028 |
| rs9368112 | 6 | chr6:19718157 | T | C | 0.529 | 0.026 |
| rs9379084 | 6 | chr6:7231843 | G | A | 0.884 | 0.075 |
| rs9383649 | 6 | chr6:153428102 | G | A | 0.403 | 0.033 |
| rs9555581 | 13 | chr13:109944192 | C | T | 0.613 | 0.03 |
| rs9563574 | 13 | chr13:58656599 | T | C | 0.827 | 0.041 |
| rs9784137 | 2 | chr2:121325908 | G | A | 0.845 | 0.061 |
| rs980183 | 2 | chr2:59311536 | G | A | 0.397 | 0.036 |
| rs9828772 | 3 | chr3:129333182 | C | G | 0.897 | 0.052 |
| rs9842137 | 3 | chr3:3649850 | T | C | 0.001 | 0.425 |
| rs9852406 | 3 | chr3:135625498 | T | C | 0.253 | 0.038 |
| rs9854769 | 3 | chr3:185520948 | G | A | 0.314 | 0.108 |
| rs9873519 | 3 | chr3:124921457 | T | C | 0.532 | 0.038 |
| rs9873618 | 3 | chr3:170733076 | G | A | 0.71 | 0.058 |
| rs987949 | 4 | chr4:85384069 | A | G | 0.01 | 0.153 |
| rs9900074 | 17 | chr17:46124326 | G | C | 0.928 | 0.055 |
| rs9912236 | 17 | chr17:77895311 | C | T | 0.754 | 0.031 |
| rs9927842 | 16 | chr16:15153717 | T | C | 0.155 | 0.038 |
| rs10097617 | 8 | chr8:95961626 | T | C | 0.469 | 0.037 |
| rs10137475 | 14 | chr14:58797953 | G | A | 0.422 | 0.026 |
| rs10145154 | 14 | chr14:79939525 | T | C | 0.222 | 0.055 |
| rs10188334 | 2 | chr2:653874 | C | T | 0.828 | 0.05 |
| rs10240790 | 7 | chr7:89880949 | G | A | 0.714 | 0.028 |
| rs10305420 | 6 | chr6:39016636 | C | T | 0.608 | 0.032 |
| rs10404726 | 19 | chr19:18834514 | C | T | 0.533 | 0.028 |
| rs10406327 | 19 | chr19:33890838 | C | G | 0.521 | 0.037 |
| rs10407429 | 19 | chr19:46157237 | G | A | 0.574 | 0.054 |
| rs10471048 | 4 | chr4:83587562 | G | C | 0.337 | 0.034 |
| rs10490871 | 3 | chr3:35667761 | G | A | 0.365 | 0.027 |
| rs1059592 | 8 | chr8:22477778 | A | G | 0.354 | 0.027 |
| rs10737818 | 1 | chr1:235542023 | G | A | 0.643 | 0.04 |
| rs10750397 | 11 | chr11:128234144 | A | G | 0.278 | 0.048 |
| rs10769936 | 11 | chr11:8654528 | C | T | 0.725 | 0.035 |
| rs10771372 | 12 | chr12:27962260 | C | T | 0.803 | 0.072 |
| rs10771813 | 12 | chr12:31367856 | C | A | 0.554 | 0.026 |
| rs10787287 | 10 | chr10:112647195 | T | C | 0.767 | 0.036 |
| rs10788575 | 10 | chr10:89768584 | A | G | 0.164 | 0.035 |
| rs10811661 | 9 | chr9:22134094 | T | C | 0.826 | 0.138 |
| rs10821311 | 9 | chr9:96943059 | A | G | 0.318 | 0.036 |
| rs10830963 | 11 | chr11:92708710 | G | C | 0.274 | 0.089 |
| rs10841868 | 12 | chr12:21781246 | G | T | 0.74 | 0.032 |
| rs10844518 | 12 | chr12:33410780 | G | A | 0.279 | 0.033 |
| rs10882891 | 10 | chr10:99059645 | C | A | 0.403 | 0.031 |

**Table S2.** Examples of food items constituting the 39 food groups used to calculate the inflammatory diet index (IDI) from the Oxford WebQ questionnaire of the UK Biobank.

| **Food groups** |  |
| --- | --- |
| **Wine** | Red wine, rose wine, white wine, fortified wine |
| **Beer** | Beer/cider |
| **Other alcohol** | Spirits, other alcohol |
| **Bread** | Sliced bread, baguette, bap, bread roll, naan bread, garlic bread, crispbread, oatcakes, other bread |
| **Butter** | Butter/margarine |
| **Starch** | White pasta, white rice, sushi, couscous |
| **Breakfast cereal** | Porridge, muesli, oat crunch, plain cereal, bran cereal, whole-wheat cereal, other cereal |
| **Bean** | Baked bean, pulses, broad bean, green bean, pea |
| **Potato** | Fried potatoes, boiled/baked potatoes, mashed potato |
| **Vegetable** | Mixed vegetable, vegetable pieces, coleslaw, side salad, avocado, beetroot, broccoli, butternut squash, cabbage/kale, carrot, cauliflower, celery, courgette, cucumber, leek, lettuce, mushroom, onion, parsnip, sweet pepper, spinach, sprouts, sweetcorn, sweet potato, fresh tomato, tinned tomato, turnip/swede, watercress, other vegetables |
| **Garlic** | Garlic |
| **Fruit** | Stewed fruit, prune, dried fruit, mixed fruit, apple, banana, berry, cherry, grapefruit, grape, mango, melon, orange, satsuma, peach/nectarine, pear, pineapple, plum, other fruit |
| **Low calorie drink** | Low calorie drink |
| **High calorie drink** | Fizzy drink |
| **Juice** | Orange juice, grapefruit juice, pure fruit/vegetable juice |
| **Smoothie** | Fruit smoothie, dairy smoothie |
| **Coffee** | Instant coffee, filtered coffee, cappuccino, latte, espresso, other coffee |
| **Tea** | Standard tea, rooibos tea, green tea, herbal tea, other tea |
| **Milk** | Milk, flavoured milk |
| **Other drink** | Other drinks |
| **Processed meat** | Sausage, bacon, ham |
| **Red meat** | Beef, pork, lamb |
| **Poultry** | Crumbed or deep-fried poultry, poultry |
| **Organ meat** | Liver |
| **Other meat** | Other meat |
| **Fish** | Tinned tuna, oily fish, breaded fish, battered fish, white fish, prawns, Lobster/crab, shellfish, other fish |
| **Cheese** | Low fat hard cheese, hard cheese, soft cheese, blue cheese, low fat cheese spread, cheese spread, cottage cheese, feta, mozzarella, goat's cheese, other cheese |
| **Egg** | Whole egg, omelette, eggs in sandwiches, Scotch egg, other egg |
| **Pastry** | Double crust pastry, single crust pastry, crumble, pizza, pancake, Scotch pancake, Yorkshire pudding, Indian snacks, croissant, Danish pastry, scone |
| **Yogurt** | Yogurt |
| **Ice cream** | Ice cream |
| **Dessert** | Milk-based pudding, other milk-based pudding, soya dessert, fruitcake, cake, doughnut, sponge pudding, cheesecake, other dessert |
| **Chocolate** | Chocolate bar, white chocolate, milk chocolate, dark chocolate, chocolate-covered raisin, chocolate sweet, chocolate-covered biscuits, chocolate biscuits |
| **Chocolate drink** | Hot chocolate |
| **Sweet** | Sweets, sweet biscuits, cereal bar, other sweets |
| **Nut** | Salted peanuts, unsalted peanuts, salted nuts, unsalted nuts, seeds |
| **Snack** | Crisp, savoury biscuits, cheesy biscuits, olives, other savoury snacks |
| **Soup** | Powdered/instant soup, canned soup, homemade soup |
| **Vegetarian protein alternative** | Vegetarian sausages/burgers, tofu, quorn, other vegetarian alternatives |

**Table S3.** Factor loadings of reduced rank regression (RRR) dietary pattern.

| **RRR dietary pattern components^a^** | **Factor loadings** |
| --- | --- |
| **Positive associations** | |
| Low calorie drink | 0.0533 |
| Red meat | 0.0468 |
| Processed meat | 0.0374 |
| High calorie drink | 0.0221 |
| Egg | 0.0202 |
| Poultry | 0.0196 |
| Potato | 0.0192 |
| Milk | 0.0187 |
| Other alcohol | 0.0183 |
| Butter | 0.0174 |
| Ice-cream | 0.0150 |
| Chocolate drink | 0.0145 |
| Organ meat | 0.0125 |
| Sweet | 0.0080 |
| Snack | 0.0060 |
| Smoothie | 0.0056 |
| Other meat | 0.0054 |
| Beer | 0.0029 |
| **Inverse associations** | |
| Starch | -0.0503 |
| Nut | -0.0431 |
| wine | -0.0429 |
| Breakfast cereal | -0.06840 |
| Fruit | -0.06637 |
| Vegetable | -0.0224 |
| Bread | -0.01136 |
| Vegetarian protein alternative | -0.0186 |
| Fish | -0.04830 |
| Dessert | -0.02280 |
| Tea | -0.0133 |
| Pastry | -0.0105 |
| Cheese | -0.03834 |
| Juice | -0.02440 |
| Coffee | -0.01433 |
| Other drink | -0.00795 |
| Bean | -0.02389 |
| Chocolate | -0.0031 |
| Garlic | -0.0029 |
| Soup | -0.0013 |
| Yogurt | -0.0004 |

^a^ The RRR dietary pattern was the first factor obtained from RRR with all 39 food groups.

**Table S4.** Food groups included in the inflammatory diet index (IDI) and Spearman correlation coefficients between individual food group with high-sensitivity C-reactive protein (hsCRP) concentrations.

| **Products** | **Spearman correlation coefficients** | **Weights ^a^** |
| --- | --- | --- |
| **Foods with anti-inflammatory potential** | |  |
| Nut | -0.07127 | -0.00301 |
| Vegetarian protein alternative | -0.04982 | -0.00082928 |
| Starch | -0.07138 | -0.00078412 |
| Breakfast cereal | -0.06840 | -0.00076784 |
| Cheese | -0.03834 | -0.00067483 |
| Dessert | -0.02280 | -0.00036392 |
| Fish | -0.04830 | -0.00031898 |
| Wine | -0.07050 | -0.00026867 |
| Bread | -0.01136 | -0.0002396 |
| Fruit | -0.06637 | -0.0001813 |
| Pastry | -0.00790 | -0.00017012 |
| Vegetable | -0.05654 | -0.0001197 |
| Soup | -0.04618 | -0.00007641 |
| Tea | -0.02812 | -0.00003361 |
| Juice | -0.02440 | -0.00003295 |
| Coffee | -0.01433 | -0.00001992 |
| **Foods with pro-inflammatory potential** | |  |
| Butter | 0.01922 | 0.00235 |
| Organ meat | 0.01129 | 0.00204 |
| Other alcohol | 0.01565 | 0.00203 |
| Processed meat | 0.04012 | 0.002 |
| Red meat | 0.04216 | 0.00131 |
| Other meat | 0.03488 | 0.00093815 |
| Ice-cream | 0.01328 | 0.00068981 |
| Poultry | 0.00754 | 0.00064264 |
| Chocolate drink | 0.00745 | 0.00036889 |
| Low calorie drink | 0.06923 | 0.00035341 |
| Milk | 0.01714 | 0.00031821 |
| Egg | 0.01564 | 0.00031512 |
| Potato | 0.02537 | 0.00031063 |
| Snack | 0.00198 | 0.00027962 |
| Sweets | 0.00139 | 0.00027053 |
| High calorie drink | 0.03392 | 0.00024219 |
| Smoothie | 0.00352 | 0.00014106 |
| Beer | 0.00537 | 0.00002603 |

^a^ Weights are regression coefficients for each IDI component obtained from the last step of the stepwise linear regression analysis.

**Table** **S5.** Relative concentrations of plasma high-sensitivity C-reactive protein (hsCRP) across tertiles of the inflammatory diet index (IDI) in test and retest groups^*^.

|  | Low | Moderate | High | *P*-trend ^c^ |
| --- | --- | --- | --- | --- |
| Test subgroup n = 130,608 |  |  |  |  |
| Basic-adjusted ^a^ | 1.00 (Reference) | 1.34 (1.01, 1.56) | 1.68 (1.28, 2.04) | 0.002 |
| Multi-adjusted ^b^ | 1.00 (Reference) | 1.28 (1.05, 1.50) | 1.57 (1.25, 1.92) | 0.003 |
| Retest subgroup n = 55,975 |  |  |  |  |
| Basic-adjusted ^a^ | 1.00 (Reference) | 1.36 (1.04, 1.62) | 1.62 (1.21, 1.97) | <0.001 |
| Multi-adjusted ^b^ | 1.00 (Reference) | 1.27 (1.04, 1.57) | 1.51 (1.16, 1.86) | <0.001 |

^*^ Values are relative concentrations (95% CIs) of hsCRP in moderate and high relative to low IDI as the reference (i.e., the ratios of the concentrations in moderate and high IDI to that in low IDI). All values were back-transformed (e^x^) because hsCRP concentrations were ln-transformed before analyses.

^a^ Adjusted for age and sex.

^b^ Adjusted for age, sex, education, Townsend deprivation index, energy intake, smoking, physical activity, body mass index, hypertension, cardiovascular disease, antidiabetic drug, and genetic risk score.

^c^ The *P*-value of the inflammatory diet index as a continuous variable.

**Table S6.** Examples of food items from the Oxford WebQ included in the 18 food groups to calculate empirical dietary inflammatory pattern (EDIP) scores.

| **Food groups** |  |
| --- | --- |
| **Processed meat** | Sausage, ham, bacon |
| **Red meat** | Beef, pork, lamb |
| **Organ meat** | Liver |
| **Other fish** | Tinned tuna, lobster/crab, shellfish, breaded fish, battered fish, white fish, prawns, other fish |
| **Other vegetables** | Celery, mushroom, sweet pepper, sweetcorn, mixed vegetables, vegetable pieces, courgette, sprouts, cucumber |
| **Refined grains** | White bread (sliced, baguette, bap, roll), naan bread, other bread, scones, croissants, white rice, white pasta, couscous, pancakes, Scotch pancakes |
| **High-energy beverages** | Fizzy drinks, squash |
| **Low-energy beverages** | Low-calorie drinks |
| **Tomatoes** | Fresh tomatoes, tinned tomatoes |
| **Beer** | Beer/cider |
| **Wine** | Red wine, white wine |
| **Tea** | Standard tea, green tea |
| **Coffee** | Instant coffee, filtered coffee, cappuccino, latte, espresso, other coffee |
| **Dark yellow vegetables** | Carrots, butternut squash, sweet potatoes |
| **Leafy green vegetables** | Spinach, lettuce, side salad, watercress |
| **Snacks** | Crisps, savoury biscuits, cheesy biscuits, oatcakes, crispbread, other savoury snacks |
| **Fruit juice** | Orange juice, grapefruit juice, pure fruit/vegetable juice |
| **Pizza** | Pizza |

**Table S7.** Basic-adjusted hazard ratios (HRs), 50th percentile differences (PDs, years), and 95% confidence intervals (95% CIs) of incident type 2 diabetes (T2D) in relation to a low-inflammatory diet in normoglycemia and prediabetes groups.

| Inflammatory Diet Index | T2D in the normoglycemia group | | |  | T2D in the prediabetes group | | |
| --- | --- | --- | --- | --- | --- | --- | --- |
|  | No. of cases | Basic-adjusted  HR (95% CI)^a^ | Basic-adjusted  50th PDs (95% CI)^a^ |  | No. of cases | Basic-adjusted  HR (95% CI)^a^ | Basic-adjusted  50th PDs (95% CI)^a^ |
| Continuous  (per 1 SD increase) | 3,348 | 1.51 (1.47, 1.57) | -2.23 (-2.48, -2.10) |  | 2,496 | 1.24 (1.19, 1.29) | -1.08 (-1.28, -0.87) |
| Categorical |  |  |  |  |  |  |  |
| High | 1,686 | 1.00 (Reference) | 0.00 (Reference) |  | 1,115 | 1.00 (Reference) | 0.00 (Reference) |
| Moderate | 1,008 | 0.62 (0.57, 0.67) | 2.66 (2.22, 3.09) |  | 769 | 0.74 (0.67, 0.81) | 1.53 (1.06, 2.00) |
| Low | 654 | 0.43 (0.39, 0.47) | 4.67 (4.17, 5.17) |  | 612 | 0.61 (0.55, 0.67) | 2.52 (2.01, 3.04) |

^a^ Adjusted for age and sex.

**Table S8.** Hazard ratios (HRs) and 95% confidence intervals (95% CIs) for the association between levels of genetic risk score and type 2 diabetes (T2D) in normoglycemia and prediabetes groups.

| Genetic Risk Score | T2D in the normoglycemia group | | | |  | T2D in the prediabetes group | | | |
| --- | --- | --- | --- | --- | --- | --- | --- | --- | --- |
|  | No. of subjects | No. of cases | Basic-adjusted HR (95% CI)^a^ | Multi-adjusted HR (95% CI)^b^ |  | No. of subjects | No. of cases | Basic-adjusted HR (95% CI)^a^ | Multi-adjusted HR (95% CI)^b^ |
| Continuous  (per 1 SD increase) | 126,203 | 3,348 | 1.71 (1.65, 1.77) | 1.54 (1.49, 1.59) |  | 16,068 | 2,496 | 1.28(1.23, 1.33) | 1.28 (1.23, 1.33) |
| Categorical (tertiles) |  |  |  |  |  |  |  |  |  |
| Low | 42,067 | 590 | 1.00 (Reference) | 1.00 (Reference) |  | 5,356 | 655 | 1.00 (Reference) | 1.00 (Reference) |
| Moderate | 42,073 | 987 | 1.70 (1.54, 1.89) | 1.60 (1.45, 1.77) |  | 5,356, | 802 | 1.24 (1.12, 1.37) | 1.26 (1.14, 1.40) |
| High | 42,063 | 1,771 | 3.15 (2.87, 3.46) | 2.59 (2.35, 2.84) |  | 5,356 | 1,039 | 1.68 (1.52, 1.84) | 1.69 (1.53, 1.86) |

^a^ Adjusted for age and sex.

^b^ Adjusted for age, sex, education, Townsend deprivation index, energy intake, smoking, physical activity, antidiabetic drug, body mass index, inflammatory diet index, hypertension, cardiovascular disease, the first 10 principal components of ancestry, and genotyping batch.

**Table S9.** Hazard ratios (HRs) and 95% confidence intervals (95% CIs) of type 2 diabetes (T2D) according to joint categories of the inflammatory diet index (IDI) and genetic risk score in normoglycemia and prediabetes groups.

| Genetic risk score | Inflammatory diet index | T2D in the normoglycemia group | | | |  | T2D in the prediabetes group | | | |
| --- | --- | --- | --- | --- | --- | --- | --- | --- | --- | --- |
|  |  | No. of subjects | No. of cases | HR  (95% CI) ^a^ | HR  (95% CI) ^b^ |  | No. of subjects | No. of cases | HR  (95% CI) ^a^ | HR  (95% CI) ^b^ |
| High | High | 14,681 | 875 | 1.00 (Reference) | 1.00 (Reference) |  | 1,855 | 436 | 1.00 (Reference) | 1.00 (Reference) |
|  | Moderate | 13,895 | 551 | 0.65 (0.58, 0.72) | 0.82 (0.74, 0.92) |  | 1,732 | 344 | 0.87 (0.76, 1.00) | 1.03 (0.90, 1.19) |
|  | Low | 13,487 | 345 | 0.44 (0.39, 0.50) | 0.69 (0.60, 0.78 |  | 1,769 | 259 | 0.63 (0.54, 0.73) | 0.85 (0.73, 0.98) |
| Moderate | High | 14,603 | 505 | 0.55 (0.50, 0.62) | 0.61 (0.55, 0.68) |  | 1,836 | 360 | 0.80 (0.70, 0.92) | 0.83 (0.72, 0.96) |
|  | Moderate | 13,738 | 288 | 0.38 (0.30, 0.39) | 0.51 (0.45, 0.59) |  | 1,770 | 237 | 0.54 (0.46, 0.63) | 0.63 (0.54, 0.74) |
|  | Low | 13,732 | 194 | 0.24 (0.20, 0.28) | 0.42 (0.36, 0.49) |  | 1,750 | 205 | 0.50 (0.42, 0.59) | 0.61 (0.52, 0.73) |
| Low | High | 14,371 | 306 | 0.34 (0.30, 0.38) | 0.37 (0.32, 0.46) |  | 1,919 | 319 | 0.48 (0.33, 0.57) | 0.59 (0.51, 0.72) |
|  | Moderate | 14,091 | 269 | 0.19 (0.16, 0.22) | 0.29 (0.25, 0.35) |  | 1,795 | 188 | 0.43 (0.36, 0.51) | 0.52 (0.44, 0.62) |
|  | Low | 13,605 | 115 | 0.14 (0.11, 0.17) | 0.26 (0.21, 0.32) |  | 1,642 | 148 | 0.37 (0.31, 0.45) | 0.49 (0.40, 0.59) |

^a^ Adjusted for age and sex.

^b^ Adjusted for age, sex, education, Townsend deprivation index, energy intake, smoking, physical activity, body mass index, hypertension, cardiovascular disease, antidiabetic drug, the first 10 principal components of ancestry, and genotyping batch.

**Table** **S10.** Hazard ratios (HRs) and 95% confidence intervals (95% CIs) for the association between low-inflammatory diet and type 2 diabetes (T2D) in normoglycemia and prediabetes groups, stratified by genetic risk score.

| Genetic risk score | Inflammatory diet index | T2D in the normoglycemia group | | | |  | T2D in the prediabetes group | | | | |
| --- | --- | --- | --- | --- | --- | --- | --- | --- | --- | --- | --- |
|  |  | No. of subjects | No. of cases | Basic-adjusted  HR (95% CI)^a^ | Multi-adjusted HR (95% CI)^a^ |  | No. of subjects | No. of cases | Basic-adjusted  HR (95% CI)^a^ | Multi-adjusted HR (95% CI)^a^ |  |
| Low | High | 14,371 | 306 | 1.00 (Reference) | 1.00 (Reference) |  | 1,919 | 319 | 1.00 (Reference) | 1.00 (Reference) |  |
|  | Moderate | 14,091 | 169 | 0.56 (0.46, 0.67) | 0.75 (0.62, 0.91) |  | 1,795 | 188 | 0.64 (0.53, 0.76) | 0.78 (0.65, 0.94) |  |
|  | Low | 13,605 | 115 | 0.41 (0.33, 0.51) | 0.67 (0.54, 0.84) |  | 1,642 | 148 | 0.45 (0.45, 0.67) | 0.72 (0.59, 0.87) |  |
| Moderate | High | 14,603 | 505 | 1.00 (Reference) | 1.00 (Reference) |  | 1,836 | 360 | 1.00 (Reference) | 1.00 (Reference) |  |
|  | Moderate | 13,738 | 288 | 0.61 (0.52, 0.70) | 0.88 (0.74, 0.98) |  | 1,770 | 237 | 0.66 (0.56, 0.78) | 0.76 (0.64, 0.90) |  |
|  | Low | 13,732 | 194 | 0.42 (0.36, 0.50) | 0.72 (0.60, 0.85) |  | 1,750 | 205 | 0.62 (0.52, 0.73) | 0.82 (0.68, 0.98) |  |
| High | High | 14,681 | 875 | 1.00 (Reference) | 1.00 (Reference) |  | 1,855 | 436 | 1.00 (Reference) | 1.00 (Reference) |  |
|  | Moderate | 13,895 | 551 | 0.65 (0.58, 0.72) | 0.83 (0.74, 0.92) |  | 1,732 | 344 | 0.88 (0.66, 0.95) | 0.91 (0.78, 0.98) |  |
|  | Low | 13,487 | 345 | 0.44 (0.39, 0.50) | 0.66 (0.58, 0.76) |  | 1,769 | 259 | 0.63 (0.54, 0.74) | 0.78 (0.64, 0.91) |  |

^a^ Adjusted for age and sex.

^b^ Adjusted for age, sex, education, Townsend deprivation index, energy intake, smoking, physical activity, body mass index, and antidiabetic drug, hypertension, cardiovascular disease, the first 10 principal components of ancestry, and genotyping batch.

**Table S11.** Additive interaction between genetic risk score (GRS) and inflammatory diet index (IDI) for type 2 diabetes (T2D) in normoglycemia and prediabetes groups.

| Genetic risk score | Inflammatory diet index | T2D in the normoglycemia group | | | |  | T2D in the prediabetes group | | | |
| --- | --- | --- | --- | --- | --- | --- | --- | --- | --- | --- |
|  |  | No. of subjects | No. of cases | Basic-adjusted  HR (95% CI)^a^ | Multi-adjusted HR (95% CI)^b^ |  | No. of subjects | No. of cases | Basic-adjusted  HR (95% CI)^a^ | Multi-adjusted HR (95% CI)^b^ |
| Low | Low | 27,696 | 284 | 1.00 (Reference) | 1.00 (Reference) |  | 3,437 | 336 | 1.00 (Reference) | 1.00 (Reference) |
|  | Moderate/High | 14,371 | 306 | 2.05 (1.74, 2.41) | 1.44 (1.22, 1.69) |  | 1,919 | 319 | 1.67 (1.44, 1.95) | 1.35 (1.16, 1.58) |
| Moderate  /High | Low | 54,852 | 1,378 | 2.53 (2.23, 2.89) | 2.24 (1.97, 2.54) |  | 7,021 | 1,045 | 1.57 (1.39, 1.78) | 1.58 (1.40, 1.79) |
|  | Moderate/High | 29,284 | 1,380 | 4.69 (4.13, 5.33) | 2.86 (2.51, 3.26) |  | 3,691 | 796 | 2.25 (1.98, 2.56) | 1.82 (1.59, 2.07) |

^a^ Adjusted for age and sex.

^b^ Adjusted for age, sex, education, Townsend deprivation index, energy intake, smoking, physical activity, body mass index, hypertension, cardiovascular disease, antidiabetic drug, the first 10 principal components of ancestry, and genotyping batch.

Measures of additive interaction for T2D:

1. T2D in the normoglycemia group:

Relative excess risk due to interaction: 0.184, 95% CI: 0.183, 0.186

Attributable proportion due to interaction: 0.064, 95% CI: 0.063, 0.068

Synergy index: 1.110, 95% CI: 1.108, 1.112

(2) T2D in the prediabetes group:

Relative excess risk due to interaction: -0.118, 95% CI: -0.367, 0.130

Attributable proportion due to interaction: -0.065, 95% CI: -0.201, 0.071

Synergy index: 0.873, 95% CI: 0.670, 1.137

**Table S12.** Food groups included in the inflammatory diet index (IDI) and Spearman correlation coefficients between individual food group and high-sensitivity C-reactive protein (hsCRP) concentrations among participants with at least two 24-h dietary assessments (n=114,686).

| **Products** | **Spearman correlation coefficients** | **Weights** |
| --- | --- | --- |
| **Foods with anti-inflammatory potential** | | |
| Nut | -0.07099 | -0.0016 |
| Vegetarian protein alternative | -0.05366 | -0.00052218 |
| Breakfast cereal | -0.282494 | -0.00040871 |
| Starch | -0.07074 | -0.00037007 |
| Cheese | -0.03166 | -0.00028106 |
| Fish | -0.04193 | -0.00018562 |
| Wine | -0.06426 | -0.00017642 |
| Dessert | -0.00860 | -0.00016368 |
| Fruit | -0.06307 | -0.0001077 |
| Pastry | -0.00600 | -0.00009697 |
| Vegetable | -0.04645 | -0.0000906 |
| Bread | -0.00279 | -0.00007875 |
| Other drink | -0.043699 | -0.00007365 |
| Bean | -0.00448 | -0.00006775 |
| Tea | -0.02747 | -0.00003352 |
| Juice | -0.01268 | -0.00002916 |
| Coffee | -0.00282 | -0.00002368 |
| **Foods with pro-inflammatory potential** | | |
| Other alcohol | 0.01565 | 0.00107 |
| Organ meat | 0.01710 | 0.00088651 |
| Processed meat | 0.07047 | 0.00077239 |
| Butter | 0.03131 | 0.00064831 |
| Red meat | 0.07124 | 0.00053975 |
| Ice-cream | 0.02649 | 0.0003048 |
| Egg | 0.01775 | 0.00028869 |
| Other meat | 0.00274 | 0.00025488 |
| Poultry | 0.02470 | 0.00025478 |
| Sweet | 0.00985 | 0.0002287 |
| Chocolate drink | 0.01373 | 0.00022283 |
| Low calorie drink | 0.08638 | 0.00021548 |
| Snack | 0.01217 | 0.00020425 |
| Milk | 0.02543 | 0.00019518 |
| High calorie drink | 0.04404 | 0.00013027 |
| Potato | 0.05629 | 0.00012635 |
| Smoothie | 0.00276 | 0.00005504 |

**Table S13.** Hazard ratios (HRs), 50th percentile differences (PDs, years), and 95% confidence intervals (95% CIs) of incident type 2 diabetes (T2D) in relation to empirical dietary inflammatory pattern (EDIP) score in normoglycemia and prediabetes groups.

| EDIP | T2D in the normoglycemia group | |  | T2D in the prediabetes group | |
| --- | --- | --- | --- | --- | --- |
|  | Multi-adjusted HR (95% CI)^a^ | Multi-adjusted 50^th^ PDs (95% CI) (years)**^a^** |  | Multi-adjusted HR (95% CI)^a^ | Multi-adjusted 50^th^ PDs (95% CI) (years)**^a^** |
| Continuous (per 1 SD) | 1.23 (1.16, 1.34) | -0.96 (-1.17, -0.83) |  | 1.04 (1.01, 1.18) | -0.29 (-0.48, -0.09) |
| Categorical (tertiles) |  |  |  |  |  |
| High | 1.00 (Reference) | 0.00 (Reference) |  | 1.00 (Reference) | 0.00 (Reference) |
| Moderate | 0.79 (0.71, 0.92) | 1.24 (0.75, 1.69) |  | 0.91 (0.76, 0.96) | 0.58 (0.23, 1.07) |
| Low | 0.63 (0.59, 0.73) | 2.21 (1.73, 2.84) |  | 0.86 (0.81, 0.98) | 1.01 (0.48, 1.53) |

^a^ Adjusted for age, sex, education, Townsend deprivation index, energy intake, smoking, physical activity, body mass index, hypertension, cardiovascular disease, antidiabetic drug, and genetic risk score.

**Table S14.** Hazard ratios (HRs), 50th percentile differences (PDs, years), and 95% confidence intervals (95% CIs) of incident type 2 diabetes (T2D) in relation to a low-inflammatory diet in normoglycemia and prediabetes groups after excluding missing values for covariates.

| Inflammatory Diet Index | T2D in the normoglycemia group | |  | T2D in the prediabetes group | |
| --- | --- | --- | --- | --- | --- |
|  | Multi-adjusted HR (95% CI)^a^ | Multi-adjusted 50^th^ PDs (95% CI) (years)**^a^** |  | Multi-adjusted HR (95% CI)^a^ | Multi-adjusted 50^th^ PDs (95% CI) (years)**^a^** |
| Continuous (per 1 SD) | 1.18 (1.14, 1.22) | -0.92 (-1.12, -0.71) |  | 1.07 (1.03, 1.12) | -0.35 (-0.59, -0.12) |
| Categorical (tertiles) |  |  |  |  |  |
| High | 1.00 (Reference) | 0.00 (Reference) |  | 1.00 (Reference) | 0.00 (Reference) |
| Moderate | 0.82 (0.75, 0.89) | 1.04 (0.58, 1.50) |  | 0.87 (0.79, 0.96) | 0.69 (0.19, 1.19) |
| Low | 0.68 (0.62, 0.75) | 2.07 (1.51, 2.63) |  | 0.81 (0.73, 0.90) | 1.08 (0.54, 1.63) |

^a^ Adjusted for age, sex, education, Townsend deprivation index, energy intake, smoking, physical activity, body mass index, hypertension, cardiovascular disease, antidiabetic drug, and genetic risk score.

**Table** **S15.** Hazard ratios (HRs) and 95% confidence intervals (95% CIs) for the association between low-inflammatory diet and type 2 diabetes (T2D) in normoglycemia and prediabetes groups, stratified by sex.

| Sex | Inflammatory diet index | T2D in the normoglycemia group | | | |  | T2D in the prediabetes group | | | | |
| --- | --- | --- | --- | --- | --- | --- | --- | --- | --- | --- | --- |
|  |  | No. of subjects | No. of cases | Basic-adjusted  HR (95% CI)^a^ | Multi-adjusted HR (95% CI)^b^ |  | No. of subjects | No. of cases | Basic-adjusted  HR (95% CI)^a^ | Multi-adjusted HR (95% CI)^b^ |  |
| Female | High | 21,122 | 537 | 1.00 (Reference) | 1.00 (Reference) |  | 2,492 | 372 | 1.00 (Reference) | 1.00 (Reference) |  |
|  | Moderate | 23,258 | 383 | 0.61 (0.54, 0.70) | 0.81 (0.71, 0.93) |  | 2,878 | 293 | 0.65 (0.56, 0.76) | 0.84 (0.70, 0.95) |  |
|  | Low | 24,573 | 261 | 0.41 (0.35, 0.47) | 0.65 (0.56, 0.76) |  | 3,050 | 267 | 0.56 (0.48, 0.66) | 0.82 (0.70, 0.97) |  |
| Male | High | 22,533 | 1,149 | 1.00 (Reference) | 1.00 (Reference) |  | 3,118 | 743 | 1.00 (Reference) | 1.00 (Reference) |  |
|  | Moderate | 18,466 | 625 | 0.62 (0.56, 0.68) | 0.82 (0.74, 0.91) |  | 2,419 | 476 | 0.77 (0.70, 0.88) | 0.90 (0.80, 0.98) |  |
|  | Low | 16,251 | 393 | 0.44 (0.40, 0.50) | 0.69 (0.61, 0.77) |  | 2,111 | 345 | 0.64 (0.56, 0.73) | 0.80 (0.70, 0.91) |  |

^a^ Adjusted for age.

**^b^** Adjusted for age, sex, education, Townsend deprivation index, energy intake, smoking, physical activity, body mass index, hypertension, cardiovascular disease, antidiabetic drug, and genetic risk score.

**Table** S**16.** Hazard ratios (HRs) and 95% confidence intervals (95% CIs) for the association between low-inflammatory diet and type 2 diabetes (T2D) in normoglycemia and prediabetes groups, stratified by age.

| Age | Inflammatory diet index | T2D in the normoglycemia group | | | |  | T2D in the prediabetes group | | | | |
| --- | --- | --- | --- | --- | --- | --- | --- | --- | --- | --- | --- |
|  |  | No. of subjects | No. of cases | Basic-adjusted  HR (95% CI)^a^ | Multi-adjusted HR (95% CI)^b^ |  | No. of subjects | No. of cases | Basic-adjusted  HR (95% CI)^a^ | Multi-adjusted HR (95% CI)^b^ |  |
| ≤60 | High | 29,027 | 866 | 1.00 (Reference) | 1.00 (Reference) |  | 2,733 | 521 | 1.00 (Reference) | 1.00 (Reference) |  |
|  | Moderate | 26,311 | 452 | 0.60 (0.54, 0.67) | 0.85 (0.76, 0.96) |  | 2,302 | 289 | 0.66 (0.58, 0.77) | 0.87 (0.75, 0.98) |  |
|  | Low | 27,102 | 285 | 0.38 (0.33, 0.44) | 0.66 (0.58, 0.77) |  | 2,347 | 229 | 0.53 (0.45, 0.62) | 0.78 (0.67, 0.92) |  |
| >60 | High | 14,628 | 820 | 1.00 (Reference) | 1.00 (Reference) |  | 2,877 | 594 | 1.00 (Reference) | 1.00 (Reference) |  |
|  | Moderate | 15,413 | 556 | 0.66 (0.59, 0.74) | 0.81 (0.73, 0.91) |  | 2,995 | 480 | 0.80 (0.71, 0.90) | 0.88 (0.78, 0.99) |  |
|  | Low | 13,722 | 556 | 0.50 (0.44, 0.56) | 0.69 (0.61, 0.79) |  | 2,814 | 383 | 0.68 (0.60, 0.77) | 0.83 (0.73, 0.95) |  |

^a^ Adjusted for sex.

**^b^** Adjusted for age, sex, education, Townsend deprivation index, energy intake, smoking, body mass index, physical activity, hypertension, cardiovascular disease, antidiabetic drug, and genetic risk score.

**Table S17.** Hazard ratios (HRs) and 95% confidence intervals (95% CIs) for the association between low-inflammatory diet and type 2 diabetes (T2D) in normoglycemia and prediabetes groups, stratified by physical activity.

| Physical activity | Inflammatory diet index | T2D in the normoglycemia group | | | |  | T2D in the prediabetes group | | | | |
| --- | --- | --- | --- | --- | --- | --- | --- | --- | --- | --- | --- |
|  |  | No. of subjects | No. of cases | Basic-adjusted  HR (95% CI)^a^ | Multi-adjusted HR (95% CI)^b^ |  | No. of subjects | No. of cases | Basic-adjusted  HR (95% CI)^a^ | Multi-adjusted HR (95% CI)^b^ |  |
| Unfavorable | High | 5,244 | 330 | 1.00 (Reference) | 1.00 (Reference) |  | 790 | 180 | 1.00 (Reference) | 1.00 (Reference) |  |
|  | Moderate | 3,940 | 145 | 0.59 (0.48, 0.71) | 0.67 (0.55, 0.87) |  | 608 | 125 | 0.90 (0.71, 0.96) | 0.94 (0.81, 0.99) |  |
|  | Low | 3,180 | 99 | 0.52 (0.41, 0.65) | 0.63 (0.53, 0.83) |  | 444 | 64 | 0.63 (0.47, 0.84) | 0.76 (0.57, 0.93) |  |
| Intermediate | High | 28,992 | 1,081 | 1.00 (Reference) | 1.00 (Reference) |  | 3,747 | 750 | 1.00 (Reference) | 1.00 (Reference) |  |
|  | Moderate | 28,228 | 673 | 0.63 (0.57, 0.70) | 0.85 (0.77, 0.94) |  | 3,577 | 489 | 0.69 (0.61, 0.78) | 0.84 (0.72, 0.92) |  |
|  | Low | 27,213 | 406 | 0.41 (0.37, 0.46) | 0.66 (0.59, 0.75) |  | 3,492 | 428 | 0.63 (0.56, 0.71) | 0.81 (0.70, 0.89) |  |
| Favorable | High | 9,419 | 275 | 1.00 (Reference) | 1.00 (Reference) |  | 1,073 | 185 | 1.00 (Reference) | 1.00 (Reference) |  |
|  | Moderate | 9,556 | 190 | 0.67 (0.56, 0.81) | 0.88 (0.73, 0.98) |  | 1,112 | 155 | 0.81 (0.65, 0.96) | 0.95 (0.78, 0.97) |  |
|  | Low | 10,431 | 149 | 0.51 (0.42, 0.62) | 0.76 (0.62, 0.93) |  | 1,225 | 120 | 0.58 (0.46, 0.73) | 0.76 (0.60, 0.96) |  |

^a^ Adjusted for age and sex.

**^b^** Adjusted for age, sex, education, Townsend deprivation index, energy intake, smoking, body mass index, hypertension, cardiovascular disease, antidiabetic drug, and genetic risk score.

**Table S18.** Mediating effects of high-sensitivity C-reactive protein (hsCRP) in the association between inflammatory diet index (IDI) and incident type 2 diabetes.

|  | **Fully adjusted *β*-coefficient (95% CI)** ^*^ | ***P*-value** |
| --- | --- | --- |
| **Total effect of IDI** | 0.0038 (0.0029, 0.0047) | <0.001 |
| **Mediator, hsCRP** |  |  |
| **Direct effect** | 0.0035 (0.0026, 0.0045) | <0.001 |
| **Mediating effect** | 0.00027 (0.00021, 0.00029) | <0.001 |
| **Percent mediation** | 7.10% |  |

^a^ Adjusted for age, sex, education, Townsend deprivation index, energy intake, smoking, physical activity, body mass index, hypertension, cardiovascular disease, antidiabetic drug, and genetic risk score.

**0.0035 (0.0026-0.0045)** ^*^

**0.0865 (0.0785-0.0945)** ^*^

**0.0031 (0.0025-0.0036)** ^*^

**IDI**

**0.0038 (0.0029-0.0047)**^*^

**T2D**

**hsCRP**

**Mediation：7.10%**

**IDI**

**T2D**

**Figure S1.** Mediating effects of high-sensitivity C-reactive protein (hsCRP) in the association between inflammatory diet index (IDI) and incident type 2 diabetes (T2D).

Model was adjusted for adjusted for age, sex, education, Townsend deprivation index, energy intake, smoking, physical activity, body mass index, hypertension, cardiovascular disease, and antidiabetic drug.

^*^ *p* < 0.001.
